# BA.1, BA.2 and BA.2.75 variants show comparable replication kinetics, reduced impact on epithelial barrier and elicit cross-neutralizing antibodies

**DOI:** 10.1371/journal.ppat.1011196

**Published:** 2023-02-24

**Authors:** Janmejay Singh, Anbalagan Anantharaj, Aleksha Panwar, Chitra Rani, Monika Bhardwaj, Parveen Kumar, Partha Chattopadhyay, Priti Devi, Ranjeet Maurya, Pallavi Mishra, Anil Kumar Pandey, Rajesh Pandey, Guruprasad R. Medigeshi

**Affiliations:** 1 Bioassay Laboratory and Clinical and Cellular Virology Laboratory, Translational Health Science and Technology Institute, Faridabad, Haryana, India; 2 INtegrative GENomics of HOst-PathogEn (INGEN-HOPE) laboratory, CSIR-Institute of Genomics and Integrative Biology, Delhi, India; 3 Academy of Scientific and Innovative Research (AcSIR), Ghaziabad, India; 4 Employees State Insurance Corporation Medical College and Hospital, Faridabad, Haryana, India; The Peter Doherty Institute and Melbourne University, AUSTRALIA

## Abstract

The Omicron variant of SARS-CoV-2 is capable of infecting unvaccinated, vaccinated and previously-infected individuals due to its ability to evade neutralization by antibodies. With multiple sub-lineages of Omicron emerging in the last 12 months, there is inadequate information on the quantitative antibody response generated upon natural infection with Omicron variant and whether these antibodies offer cross-protection against other sub-lineages of Omicron variant. In this study, we characterized the growth kinetics of Kappa, Delta and Omicron variants of SARS-CoV-2 in Calu-3 cells. Relatively higher amounts infectious virus titers, cytopathic effect and disruption of epithelial barrier functions was observed with Delta variant whereas infection with Omicron sub-lineages led to a more robust induction of interferon pathway, lower level of virus replication and mild effect on epithelial barrier. The replication kinetics of BA.1, BA.2 and BA.2.75 sub-lineages of the Omicron variant were comparable in cell culture and natural infection in a subset of individuals led to a significant increase in binding and neutralizing antibodies to the Delta variant and all the three sub-lineages of Omicron but the level of neutralizing antibodies were lowest against the BA.2.75 variant. Finally, we show that Cu^2+^, Zn^2+^ and Fe^2+^ salts inhibited *in vitro* RdRp activity but only Cu^2+^ and Fe^2+^ inhibited both the Delta and Omicron variants in cell culture. Thus, our results suggest that high levels of interferons induced upon infection with Omicron variant may counter virus replication and spread. Waning neutralizing antibody titers rendered subjects susceptible to infection by Omicron variants and natural Omicron infection elicits neutralizing antibodies that can cross-react with other sub-lineages of Omicron and other variants of concern.

## Author summary

SARS-CoV-2 variants are under selection pressure to evolve in the presence of humoral and T-cell immunity from vaccination and or natural infections. Newer variants of the SARS-CoV-2 Omicron and its sub-lineages have emerged in the past few months with each of the variants harboring additional mutations primarily in the spike region to enhance infectivity and transmission and also to escape neutralization from pre-existing antibodies. We show here that the BA.1, BA.2 and BA.2.75 sub-lineages of the Omicron variant have comparable or relatively better capacity for viral entry. However, despite comparable kinetics of replication, the omicron sub-lineages have compromised ability to disrupt the epithelial barrier in lung epithelial cell lines. These newer variants have accumulated several unique mutations in the spike region to escape from humoral immunity and our data suggests that BA.2.75 is the most antibody evasive variant. Both the Delta and the Omicron variants were inhibited by copper and iron salts thus providing an option for supplementation trials in SARS-CoV-2 patients. Overall, the evolution of SARS-CoV-2 is geared towards optimizing viral entry, replication and immune evasion which may favor attaining endemic status due to reduced pathogenicity.

## Introduction

Close to 17 million cases of COVID-19 was reported from India during the second wave of SARS-CoV-2 infection from March 2021 to June 2021 overwhelming the public health infrastructure resulting in close to 40% of the >500,000 deaths that has occurred in this pandemic. The surge was due to novel SARS-CoV-2 variant from the lineage B.1.617.2 (Delta variant) which was shown to be more virulent with shorter incubation periods and ability to cause severe disease [1–4]. Many reports have shown that the neutralizing antibodies from prior infection or that elicited by some of the licensed COVID-19 vaccines show a decrease in the efficiency to neutralize the Delta variant thereby compromising vaccine efficacy against this variant [5–7]. Delta variant was the major circulating SARS-CoV-2 variant until the emergence of B.1.1.529 (Omicron) variant in November 2021 which has now replaced the Delta variant in most of the countries [8,9]. The third wave of SARS-CoV-2 infections, which was attributed to the Omicron variant of SARS-CoV-2, has resulted in milder symptoms and lower hospitalizations which is likely due to attenuated pathogenicity relative to the Delta variant [10]. In addition to the less virulent Omicron variant, increased vaccination coverage and hybrid immunity may have contributed to reduced impact of third wave in India. Subsequently, novel sub-lineages of Omicron (BA.1-BA.5 and the sub-lineages of BA.2) have emerged and are currently circulating in India and other parts of the world [11,12]. The success of Omicron variant and its sub-lineages in driving the current phase of the pandemic has been attributed to a large number of mutations leading to escape from neutralizing antibodies generated by prior infection or vaccination [13–18]. Recent reports suggest that the antibody response between the sub-lineages may not be significantly different and exposure to one of the sub-lineages offers cross-protection against other members of Omicron sub-lineage and also against past variants [19,20].

In addition to evading humoral immunity, data from animal models suggest that the success of SARS-CoV-2 variants of concern (VoCs) in driving new waves of infection can be attributed to multiple factors such as increased virulence, enhanced transmission, altered pathogenicity and replication fitness [21–26]. Therefore, efforts to characterize and understand the replication kinetics of circulating VoCs would provide information on virulence mechanisms, immune evasion properties and augment our ability to develop therapeutic strategies. In this study, we compared the replication kinetics of some of the SARS-CoV-2 VoCs namely, the Kappa and Delta variants and BA.1, BA.2, BA.2.75 sub-lineages of Omicron variant in human lung epithelial adenocarcinoma (Calu-3) cells grown on transwells and also in air-liquid interface models. We monitored the interferon response and its downstream effectors in infected cells and tested the effect of divalent cations on virus infection and RNA-dependent RNA polymerase (RdRp) activity. We show that both binding and neutralizing antibodies wane by >50% in six months in a cohort of participants with hybrid immunity. Natural exposure to the Omicron variant led to a significant increase in binding and neutralizing antibodies to BA.1 and BA.2 sub-lineages of Omicron. The levels of neutralizing antibodies were higher against the Delta variant as compared to the Omicron sub-lineages but the potency of antibodies to neutralize BA.2.75 was significantly lower compared to BA.1 and BA.2. These data suggest that Omicron infection elicits neutralizing antibodies that can cross-react with other sub-lineages of Omicron and other VoCs in people with hybrid immunity. Our results provide clues to the relative replication fitness of these VoCs and their ability to induce interferon responses and susceptibility to divalent cations and may help to develop novel strategies to counter viral replication.

## Results

### Peak COVID-19 positivity in second wave coincides with high viral load

A total of 117,434 nasopharyngeal/oropharyngeal (NP/OP) swab samples were tested for COVID-19 by RT-PCR at the bioassay laboratory of the institute from April 2020 to July 2021. Peak positivity in the year 2020 was observed in the months of June-July with 15% positivity during these months (S1A(i) Fig). Previous reports have identified B.6 lineage as one of the predominant lineages during this period circulating in India [27,28]. We have isolated few clinical isolates from B.6 lineage as reported earlier and plaque-purified one of the isolates which was used in this study as a representative of ancestral virus isolate [29]. The number of positive cases was around 10% of the total samples tested until November 2020 and dropped drastically to around 0.1% of the total tested samples in the month of February 2021 (S1A(ii) Fig). Although only 8 of the 8012 tested samples were RT-PCR positive in February 2021, 4 of these 8 samples had RT-PCR cycle threshold (Ct) values <20 and 2 with Ct values between 20–25. RT-PCR positivity jumped to 1.1% (85 out of 7558) in March with over 50% of the positive samples having Ct values of <25 clearly indicating a large number of infections with a high viral load during February and March 2021 (S1B Fig). The following months of April and May saw a sudden spike in the peak positivity rate which increased to 27.7% and 24.5% which is now termed as the "second wave" and the SARS-CoV-2 variants from B.1.617.1/2 (Kappa/Delta) lineages have been implicated in this sudden spike which overwhelmed the healthcare infrastructure leading to large scale mortality [30]. 40% of the NP/OP samples tested in April 2021 had Ct values of less than 25 which reduced to 28% of the total samples in May indicating that high viral burden could have contributed to increased transmission of the virus among the contacts during this period.

### Disruption of cellular junctions by SARS-CoV-2 Delta and Kappa variants

**Fig 1:**
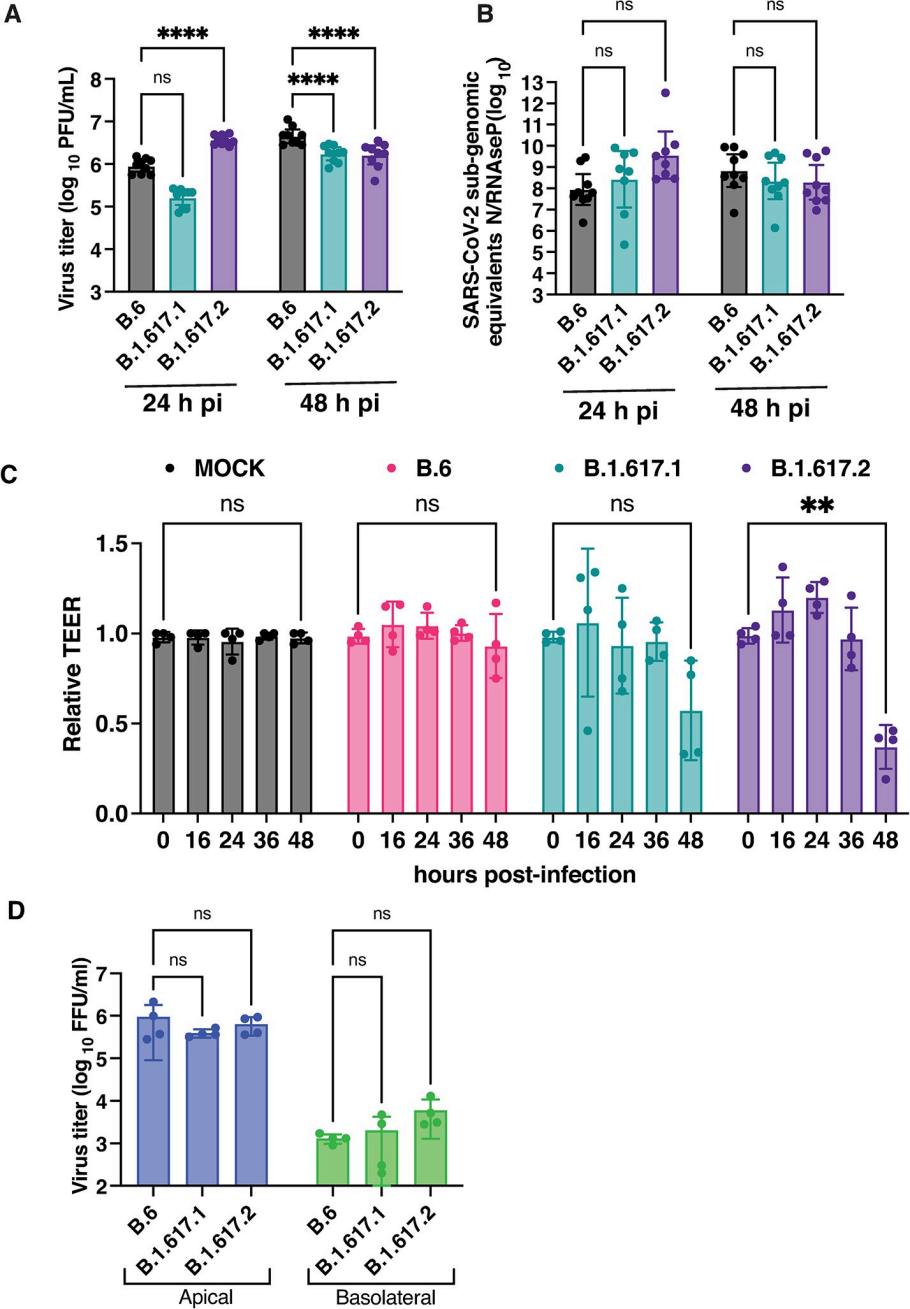
Growth curve analysis of B6, B.1.617.1, and B.1.617.2 variants in Calu-3 cells. Calu-3 cells were infected with 0.01 MOI of SARS-CoV-2 virus B.6, B.1.617.1 (Kappa), and B.1.617.2 (Delta) variants. (A) Virus titer in the culture supernatant collected at indicated time-points were estimated by plaque assay. Error bars represent geometric mean with 95% CI. (B) Sub-genomic RNA of N gene was estimated by qRT-PCR. *RNAse P* was used as internal control for normalization. Error bars represent geometric mean with 95% CI. (C) Calu-3 cells grown on transwell inserts were infected with above variants at 0.3 MOI and TEER was monitored at indicated time points. Values (Mean ± SD) are represented as relative to time zero before infection and statistical significance was determined by comparing the zero hour and 48 h readings by two-way ANOVA with Dunnett’s multiple comparisons test. (D) Viral titers were measured in supernatants at 48 h pi by focus-forming units. Data are from two or three independent experiments (Mean ± SD). ns: non-significant; ** P<0.01; **** P<0.0001.

**Fig 2:**
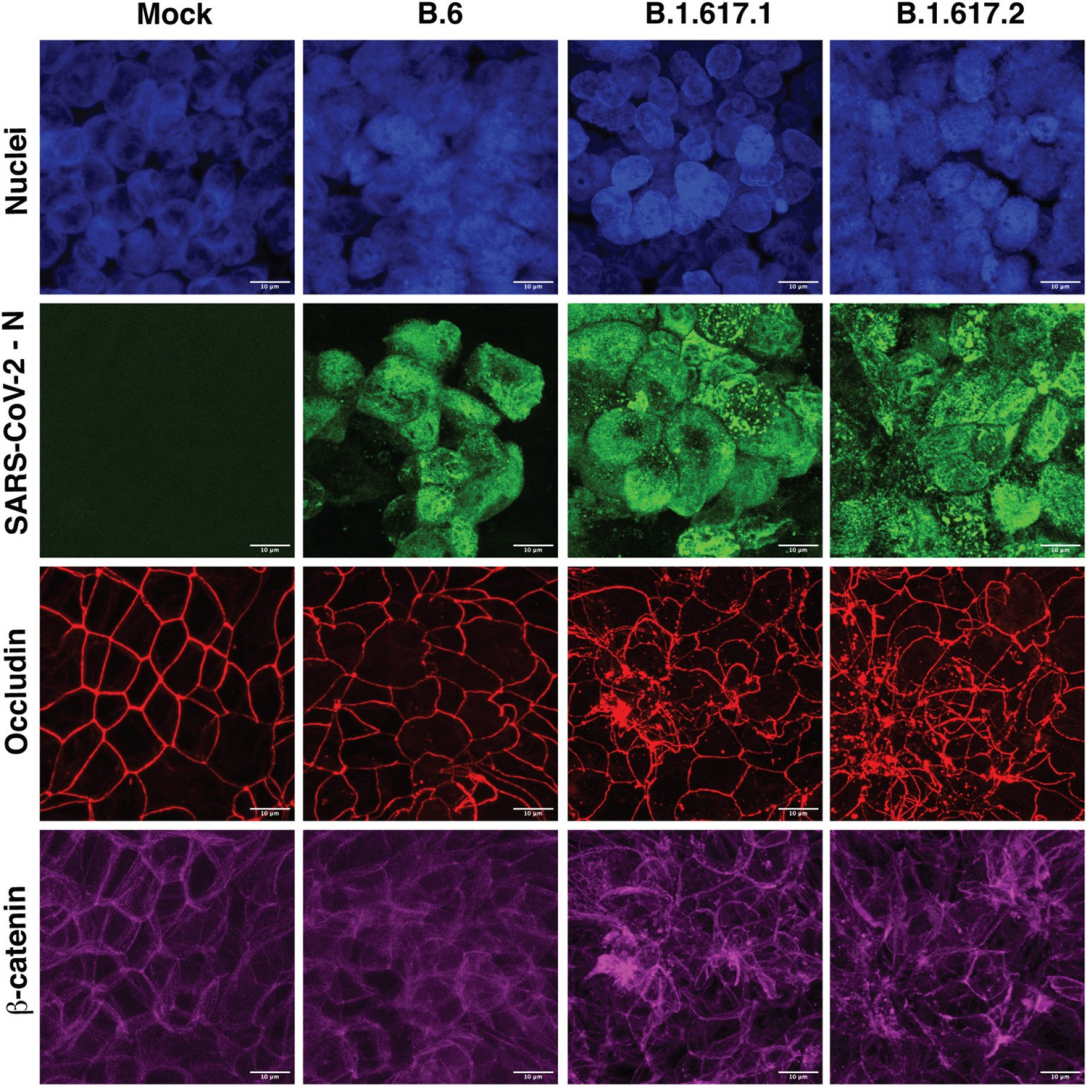
Kappa and Delta variants of SARS-CoV-2 disrupt epithelial junctions. Calu-3 cells were grown on transwell inserts and infected with indicated variants of SARS-CoV-2 at 0.3 MOI. At 48 h pi, cells were fixed and stained with occludin and β-catenin antibodies. Nucleocapsid antibody was used to visualize the SARS-CoV-2 infection. Appropriate Alexa Fluor dye-conjugated secondary antibodies were used for visualization. Nuclei were stained with DAPI. Images were captured at 100X magnification. Images were analyzed using cellSens software and Z-projection at maximum intensity images are shown in the figure. Scale bar is 10 μM.

We were able to isolate the B.1.617.1 (Kappa) and B.1.617.2 (Delta) variants from clinical samples and got the virus stocks verified by whole genome sequencing. We measured the growth kinetics of these two variants in comparison with the B.6 lineage virus. Calu-3 cells were infected with 0.01 MOI of SARS-CoV-2 from lineages B.6, B.1.617.1 and B.1.617.2 respectively. We estimated viral titers in the supernatants at 24 and 48 h pi by plaque assay. Total RNA was isolated from cells at each time point for measuring copy numbers of N gene by quantitative RT-PCR. We observed a significantly higher viral titers with B.1.617.2 variant relative to B.6 lineage virus at 24 h pi. The virus titers at 48 h pi was reduced in both the Kappa and the Delta variant samples most probably due to the increased cytopathic effect at this time point (Fig 1A). However, no difference in N gene RNA copy numbers were detected between the three isolates by RT-PCR at either of the time-points (Fig 1B). As SARS-CoV-2 was shown to disrupt tight junctions in Calu-3 and human airway epithelial cells [29,31], we further assessed the effect of infection of B.6 and B.1.617.1 and B.1.617.2 variants on cellular junctions of polarized Calu-3 cells. Cells were grown on transwell culture inserts for 21 days to allow for polarization into apical and basolateral surface and formation of permeability barrier as measured by the trans-epithelial electrical resistance (TEER). Polarized Calu-3 cells were infected with 0.3 MOI of each of the three virus isolates and the TEER was monitored for 48 hours. Cells that were either mock-infected or infected with B.6 lineage virus showed no significant change in TEER up to 48 h (Fig 1C). Epithelial barrier function was disrupted by 48 h in cells infected with either the Kappa (B.1.617.1) or the Delta (B.1.617.2) variants. However, a significant decline in the TEER values was observed for the Delta variant (Fig 1C). Surprisingly, the virus titers in either the apical or basolateral chambers were not significantly different between the three isolates (Fig 1D) at 48 h pi suggesting that the increasing cytopathogenicity of the Delta variant may be responsible for significantly higher disruption of lung epithelial barrier functions. These findings were further corroborated by confocal microscopy where transwell culture inserts were fixed at 48 h pi and stained with antibodies against SARS-CoV-2 nucleocapsid (N) protein, occludin (marker for tight junction) and β-catenin (marker for adherens junction) (Fig 2). The staining pattern of both occludin and β-catenin was comparable between mock- and B.6-infected cells. However, the same was perturbed in both the Kappa and the Delta variant infected cells and disruption of occludin staining appeared to be more drastic in cells infected with the Delta variant compared to the Kappa variant (Fig 2). Our results suggest that the Delta variant infection has relatively more deleterious effects on epithelial cell functions most probably due to increased fusogenic functions leading to syncytia formation and cell death [25].

### Infection with Omicron variant generates lower levels of infectious virus

**Fig 3:**
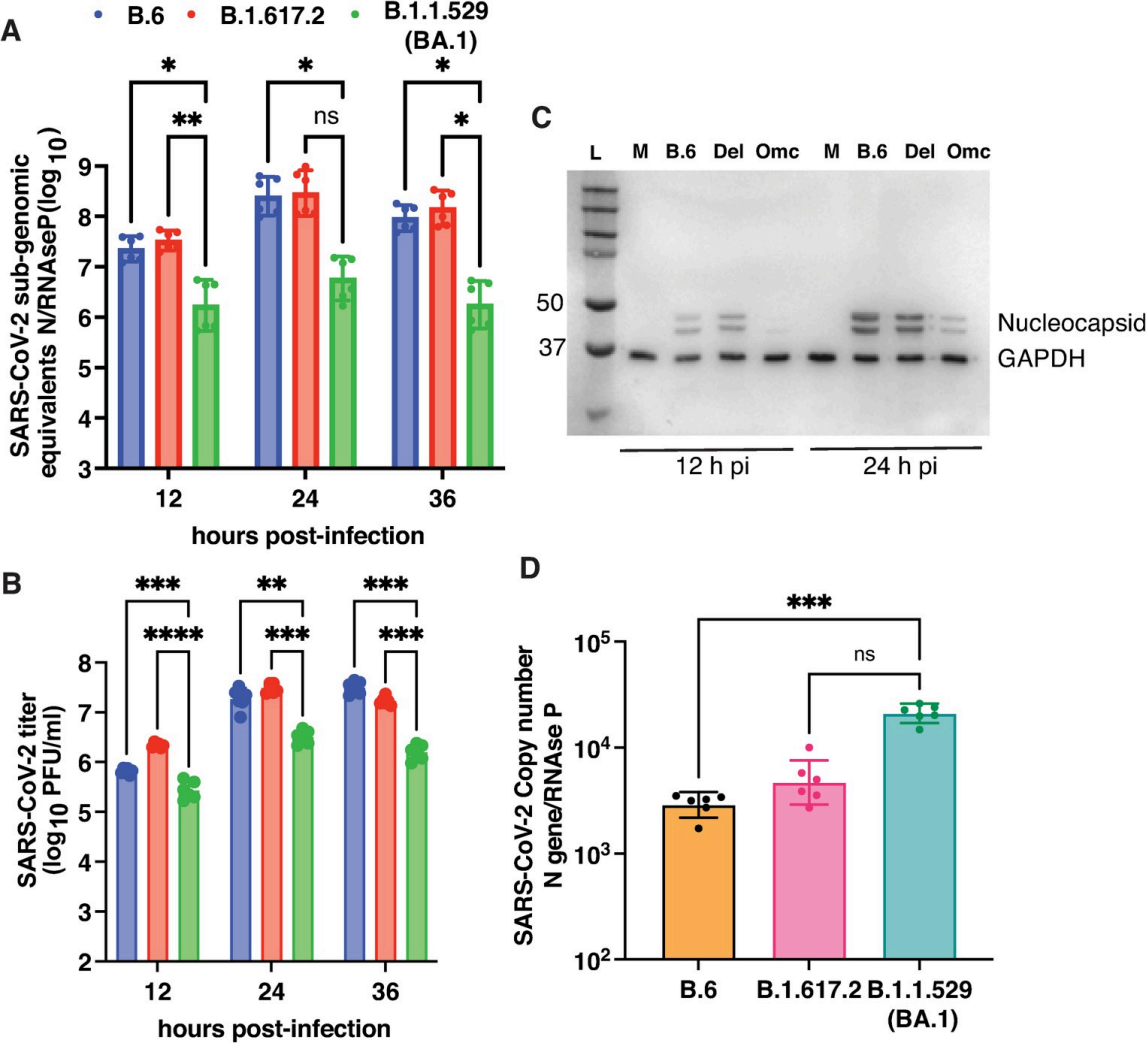
Growth kinetics of B6, B.1.617.2, and B.1.1.529 variants in Calu-3 cells. (A) Calu-3 cells were infected with above variants of SARS-CoV-2 at 0.3 MOI. Cells were collected at indicated time points and total RNA was isolated to estimate *N* gene copy numbers by RT-qPCR. *RNase P* was used as a housekeeping control for normalization. Error bars represent geometric mean with 95% CI. (B) Viral titers were measured in supernatants by plaque assay. Error bars represent geometric mean with 95% CI. Statistical significance was estimated by two-way ANOVA with Dunnett’s multiple comparisons test. (C) Western blot analysis of cell lysates prepared from infected cells at indicated time points to detect the expression of SARS-CoV-2 nucleocapsid. Glyceraldehyde 3-phosphate dehydrogenase (GAPDH) was used as a loading control. Numbers on the left indicate the size of the bands in molecular weight marker. (D) Calu-3 cells were infected at 5 MOI and the amount of internalized RNA was estimated by RT-qPCR as described above. Data are from two independent experiments. Error bars represent geometric mean with 95% CI. Statistical significance was estimated by Kruskal-Wallis test with Dunn’s multiple comparison test. ns: non-significant, * P<0.05, ** P<0.01, *** P<0.001, **** P<0.0001.

Delta variant was reported to have higher transmissibility and pathogenicity in humans and animal models [3,25,32,33] whereas Omicron variant (BA.1) showed attenuated growth [10,23,34]. We performed growth curve analysis of SARS-CoV-2 viruses from the lineages B.6 (isolate from 2020), B.1.617.2 (Delta) and B.1.1.529 (Omicron BA.1) in Calu-3 cells by infecting cells with 0.3 MOI and estimating viral genome equivalents (N gene) by qRT-PCR from infected cells and virus titers from culture supernatants by plaque assays at indicated time-points (Fig 3). We found that viral RNA replication peaked by 24 h pi for both the B.6 and the Delta variants and both these isolates had comparable viral N-gene levels by qRT-PCR at all the time points. However, the same for Omicron variant was about 70% lower as compared to the other two lineages at all the time-points (Fig 3A). Similarly, viral titers in the supernatant increased from 12 h pi to 24 h pi and the levels were maintained at 36 h pi in the case of B.6 lineage virus. However, consistent with the sub-genomic N RNA levels, the viral titers for the Omicron variant was significantly lower compared to both B.6 and the Delta variant at all the time points (Fig 3B) which is in agreement with recent reports [35,36]. These observations were further corroborated by western blot analysis performed with cell lysates prepared from infected cells collected in lysis buffer at 12 and 24 h pi where the N protein was consistently lower at both 12 and 24 h pi in cells infected with Omicron (BA.1) as compared to B.6 and Delta infection (Fig 3C). To further verify whether the lower titers observed in the Omicron (BA.1) infection is due to compromised viral entry, we performed viral entry experiments by infecting cells with 5 MOI for one hour and estimated the amount of internalized RNA by qRT-PCR. We found that, contrary to reduced viral titers and viral RNA levels in Omicron (BA.1) infection, the intracellular viral RNA levels were higher as compared to B.6 and the Delta variant suggesting that the reduced viral titers and viral RNA levels is not due to compromised viral entry into these cells (Fig 3D).

### Interferon-dependent responses and reduced barrier disruption by Omicron variant

**Fig 4:**
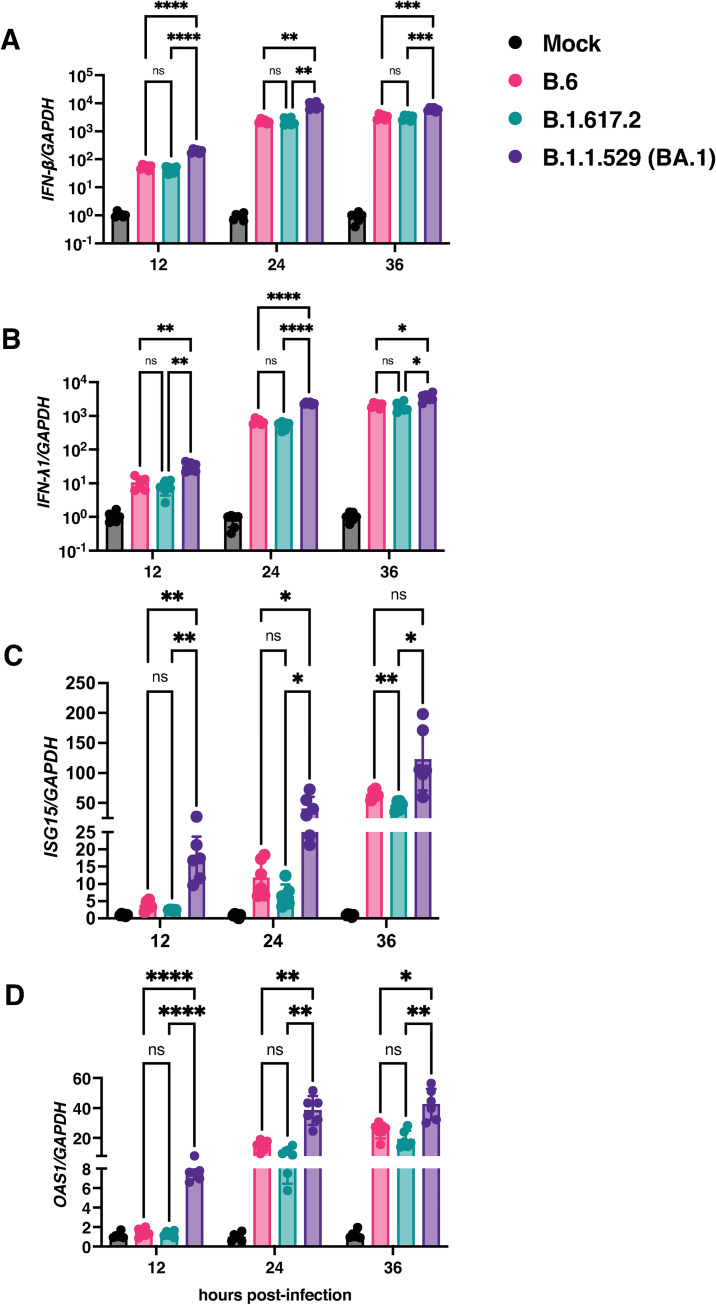
Antiviral response with B.6, B.1.617.2, and B.1.1.529 variants. Calu-3 cells were infected with indicated variants of SARS-CoV-2 at 0.3 MOI. Cells were collected at indicated time-points and total RNA was isolated. RT-qPCR was set up to determine the expression of (A) *IFN-β*. (b) *IFN-λ1*, (C) *ISG-15*, and (D) *OAS1*. *GAPDH* was used as house-keeping control for normalization. Data are from two independent experiments (Mean ± SD). Statistical significance was estimated by two-way ANOVA with Tukey’s multiple comparisons test. ns: non-significant, * P<0.05, ** P<0.01, *** P<0.001, **** P<0.0001.

Innate immune responses mediated by interferons act as the first line of defence against viral infections and SARS-CoV-2 was found to be less capable of evading the interferon response compared to SARS-CoV-1 [37,38]. Additionally, interferon induction coincided with viral clearance in young adults with mild SARS-CoV-2 infection suggesting a crucial role for antiviral response in resolving infection [39]. We tested the induction of interferon-dependent antiviral response genes in cells infected with B.6, Delta and Omicron (BA.1) lineages. Total RNA isolated from Calu-3 cells after indicated time-points post-infection were used to estimate the transcript levels of interferon pathway genes by RT-qPCR (Fig 4). We found that infection with Omicron (BA.1) variant led to significantly higher induction of interferon-β (Fig 4A) and the epithelial cell specific interferon-λ1 (Fig 4B) and the downstream effectors of interferon pathway namely, interferon-stimulated gene 15 (ISG15) (Fig 4C) and 2’-5’-oligoadenylate synthetase 1 (OAS1) (Fig 4D) at all the time-points tested suggesting that SARS-CoV-2 ancestral virus (B.6) and both the variants induce innate immune responses and lower levels of titers observed in the Omicron infected cells is due to robust induction of interferon pathway which is in agreement with previous reports [40]. To further confirm that the lower levels of replication and infection by Omicron (BA.1) variant translates to compromised ability to disrupt the epithelial barrier functions, we compared the effect of Delta and Omicron variant (BA.1) infection in an air-liquid culture model using Calu-3 cells. Cells were cultured on transwells at the air-liquid interface and infected with respective virus strains at 0.3 MOI. Trans-epithelial electrical resistance was monitored for 36 h. Although the proportion of infected cells were comparable between the Delta and the Omicron (BA.1) variant (S2A Fig), cells infected with the BA.1 variant did not show any obvious syncytia. We observed a gradual insignificant decline in the TEER values after 12 h pi in cells infected with the Omicron (BA.1) variant and, by 36 h pi, barrier integrity was compromised by 50% relative to mock-infected cells further confirming the minimal effect of this variant on epithelial barrier functions and pathogenicity as observed in animal studies by other groups [23,34]. However, infection with Delta variant led to drastic reduction of >80% in TEER values after 24 h pi suggesting rapid induction of cell death and disruption of epithelial barrier (S2B Fig). We collected culture supernatants from the basolateral chamber for measuring viral titers. In contrast to the total viral titers observed in the culture supernatants of submerged cells (Fig 3), the basolateral viral titers in the ALI model were comparable between the Omicron (BA.1) and Delta variant suggesting comparable transcytosis of both these variants but significantly higher cytopathic effect of the Delta variant as compared to the Omicron (BA.1) variant (S2C Fig). Disruption of cellular junctions was further confirmed by staining the cells with antibodies against SARS-CoV-2 N, occludin and β-catenin. As expected from the TEER values, infection with the Delta variant showed large syncytia formation as visualized by N-positive cells and disruption of and reduction in occludin and β-catenin staining (S2D Fig).

### Comparable replication kinetics of BA.1 and BA.2 sub-lineages

**Fig 5:**
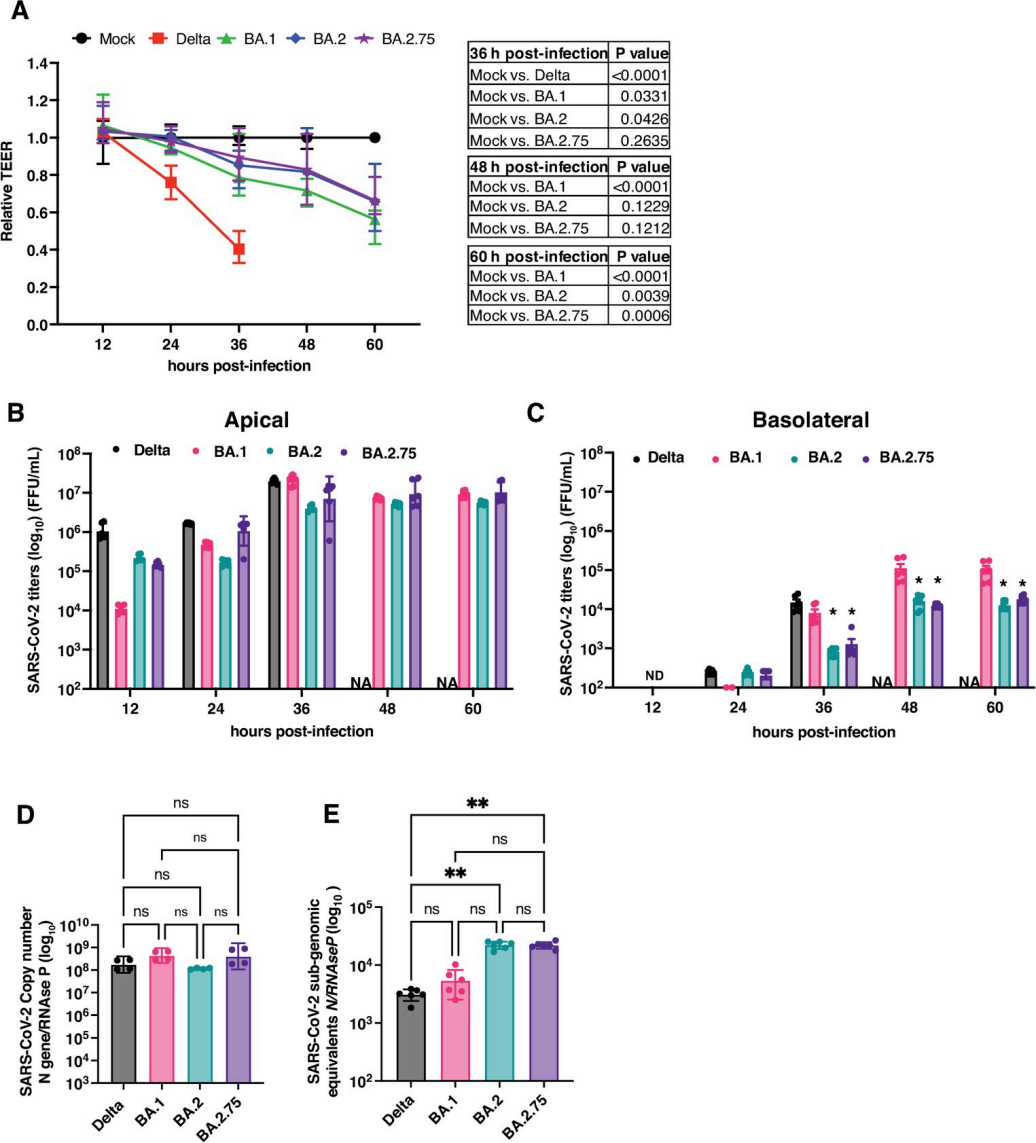
Sub-lineages of Omicron variant have milder effects on epithelial junctions. Calu-3 cells were grown on transwell inserts under air-liquid interface (ALI) conditions. Cells were infected with the indicated SARS-CoV-2 variants at 0.3 MOI. (A) Graph indicates TEER values relative to mock infection at indicated time points from two independent experiments (Mean and error with range). Statistical significance was estimated by two-way ANOVA with Tukey’s multiple comparisons test. Transwells with delta variant were discontinued at 36 h pi and therefore are indicated as NA at later points. (B) Viral titers in apical and (C) basolateral supernatants collected at indicated time points was estimated by focus-forming unit assay. Error bars represent geometric mean with 95% confidence intervals. ND: Not detected. * indicates P <0.05 relative to BA.1 sub-lineage obtained by two-way ANOVA with Dunnett’s multiple comparison test. (D) Viral RNA levels in 1000 PFU of stock viruses of indicated SARS-CoV-2 variants was quantitated by qRT-PCR. (E) Calu-3 cells were infected with 10^5^ genome equivalents of indicated SARS-CoV-2 variants and the amount of internalized RNA was estimated by RT-qPCR 1 h pi. Data are from two independent experiments. Error bars represent geometric mean with 95% CI. Statistical significance was estimated by Kruskal-Wallis test with Dunn’s multiple comparison test. ns: non-significant, * P<0.05, ** P<0.01.

SARS-CoV-2 sequencing data from the National Capital Region (NCR), our study site, showed co-occurrence of B.1.617.2 (Delta) and various AY.* (Delta plus) lineages during the months of September 2021 to December 2021, wherein all the AY lineages were more in circulation than the parent B.1.617.2 lineage (S3 Fig). It is important to note that a rise in the cases of Omicron lineages (B.1.529, BA.1 and BA.2) was observed from December 2021 onwards. This is concomitant with the arrival of Omicron variant in India and a gradual decrease in circulation of Delta lineage. Delta variant was superseded by Omicron by January 2022. Within Omicron lineages, cases of BA.2 sub-lineage increased/spread at a faster rate compared to other Omicron lineages (BA.1 and B.1.529) (S3 Fig) which is similar to reports from other countries. This is consistent with the observations that BA.2 lineage has increased rates of transmission [41,42]. As this manuscript was under review, newer sub-variants of BA.2 lineage continued to emerge and BA.2.75 was the predominant variant circulating in India after June 2022 (S4 Fig) [43]. We isolated BA.2 and BA.2.75 from symptomatic, RT-PCR-confirmed COVID-19 patients. To test the effect of sub-lineages of Omicron on the epithelial barrier functions, we compared the effect of Delta and BA.1, BA.2 and BA.2.75 sub-lineages of Omicron variant in the ALI model using Calu-3 cells. Cells were cultured on transwells at the air-liquid interface and infected with respective virus strains at 0.3 MOI. Trans-epithelial electrical resistance was monitored for 60 h but the delta variant samples were stopped at 36 h pi due to extensive cell death. Similar to our previous observation (Fig 2B), by 36 h pi, barrier integrity was compromised by >60% in cells infected with the Delta variant relative to mock-infected cells (Fig 5A). However, only the BA.1 sub-lineages of Omicron showed a significant reduction in the TEER values compared to mock infection (Fig 5A) at 36 and 48 h pi. It was only by 60 h pi that the BA.2 and BA.2.75 sub-lineages matched the TEER values of BA.1. These results suggest that the BA.2 and BA.2.75 sub-lineages are further compromised in their ability to disrupt epithelial barriers. To verify whether this effect is due to compromised infectivity of the BA.2 and BA.2.75 sub-variants, we estimated viral titers in the apical and basolateral supernatants starting from 12 h pi to 60 h pi. As the TEER values dropped drastically by 36 h pi due to extensive cell death, the transwells infected with the Delta variant was terminated at 36 h pi. Compared to the Delta variant, the viral titers of BA.1 sub-lineage in the apical culture supernatants was 100-fold lower whereas the titers of both BA.2 and BA.2.75 sub-lineages were about one order of magnitude higher than the BA.1 sub-lineage. No virus was detected in the basolateral chamber at 12 h pi. The viral titers increased with time and by 36 h pi, the viral titers in the apical supernatant for both the Delta and BA.1 variants were comparable whereas the virus titers for both BA.2 and BA.2.75 were about 10-fold lower (Fig 5B). Minimal levels of virus, barely above the detection limit of the assays, was detected in the basolateral chamber at 24 h pi onwards. The virus titers for both the Delta variant and the BA.1 variant was comparable in the basolateral chamber which correlated with significant drop in the TEER values (Fig 5C). Similar to virus titers in the apical chamber, both BA.2 and BA.2.75 titers in the basolateral chamber was significantly lower than the BA.1 sub-lineage up to 60 h pi suggesting that despite similar infectivity (as observed by the virus titers in the apical supernatants), BA.2 and BA.2.75 sub-lineages show a further delay in disruption of epithelial barriers in the ALI model (Fig 5A). The viral titers at 12 h pi in the apical supernatants showed significant differences between different VoCs despite the multiplicity of infection being similar. Therefore, we next verified whether the observed difference was due to any variation in the amount of viral genome equivalents per plaque forming unit (PFU) between these lineages. We isolated viral RNA from 1000 PFU of virus stocks of each of the lineages and estimated the copy numbers of viral genome. We found no significant difference in the number of genome equivalents/PFU between the four SARS-CoV-2 lineages (Fig 5D). Therefore, the enhanced titers observed at early time points in BA.2 and BA.2.75 is not due to technical differences but could be due to variabilities in the entry processes. Recent reports showed that the affinity of the receptor binding domain of BA.2.75 VoC was significantly higher than the other sub-lineages of the Omicron variant [43]. To further verify whether BA.2.75 has enhanced ability to enter cells, we performed viral entry assays by infecting cells with 10^5^ copies of viral RNA/cell for one hour. Intracellular RNA was isolated one hour post-infection and the amount of internalized viral RNA was quantitated by qRT-PCR. As observed previously in Fig 3, there was no difference in the internalized viral genome equivalents between Delta and BA.1 variants. However, the BA.2 and BA.2.75 sub-lineages showed a significantly higher levels of intracellular viral RNA as compared to the Delta variant suggesting a more efficient viral entry (Fig 5E).

### Variant cross-neutralizing antibodies are elicited upon natural exposure to Omicron

**Fig 6:**
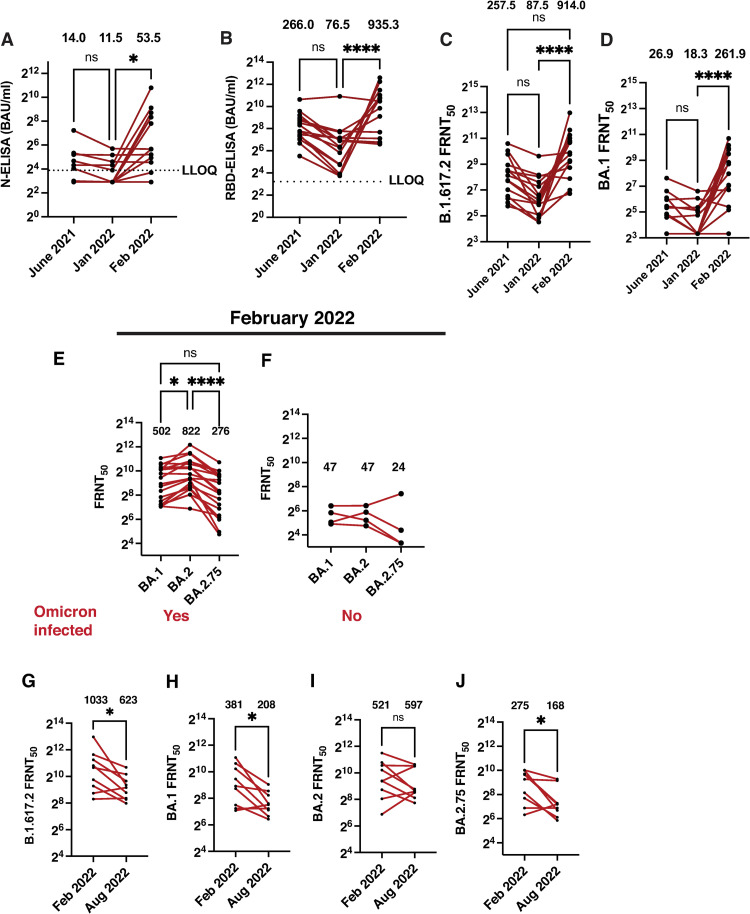
Omicron infection induces cross-protective antibodies. (A) Binding antibodies to nucleocapsid (N) and (B) RBD were estimated by quantitative ELISA for the three time-points post-vaccination as indicated (n = 15). (C) Neutralizing antibody titers to Delta variant and (D) Omicron variant was determined in the same samples by FRNT assay. Statistical significance was estimated by Kruskal-Wallis test with Dunn’s multiple comparison test. ns: not significant. * P<0.05, **** P<0.0001. (E) Neutralizing antibody titers to the BA.1, BA.2 and BA.2.75 sub-lineages were determined in samples from participants that were infected with Omicron variant in early 2022 (n = 20) and (F) indicates the same in individuals unexposed to the Omicron variants (n = 4). Numbers indicate the geometric mean NT50 values. Statistical significance was estimated by paired analysis using Friedman test with Dunn’s multiple comparison test. ns: not significant. * P<0.05, **** P<0.0001. Paired analysis for neutralizing antibodies in six month follow-up convalescent samples which were tested for neutralization of (G) Delta variant, (H) BA.1 (I) BA.2 and (J) BA.2.75. P values were obtained by Wilcoxon matched-pairs signed rank test. * P<0.05. ns: Not significant.

To further verify the potential of antibodies from vaccinated individuals to neutralize both BA.1 and BA.2 lineages, we enrolled 15 subjects (Median age 29; 5 Female, 10 Male) who were vaccinated with ChAdOx1 nCoV-19 vaccine in May 2021. We collected samples at three time points: i) first sample at one month after complete vaccination (June 2021), ii) a follow-up sample after 6 months (Dec 2021/Jan 2022) and iii) a third sample during the ongoing third wave of SARS-CoV-2 infection (Feb 2022). We performed quantitative nucleocapsid (N) and RBD ELISA [19] to monitor antibody levels in samples from the three bleeds. Six of the 15 samples had detectable levels of N antibodies (cut off: 15 BAU/mL) post-vaccination suggesting that these subjects had hybrid immunity (infection + vaccination) as the ChAdOx1 nCoV-19 vaccine does not generate N antibodies. The geometric mean titer (GMT) of N-ELISA antibodies reduced from 14.0 (95% CI: 8.4, 23.1) in June 2021 to 11.5 (95% CI: 7.9, 16.6) in Dec 2021/Jan 2022 with only five positive samples. However, the samples collected during the Omicron surge in Feb 2022 showed a significant increase in N antibodies with a GMT of 53.5 (95% CI: 19.6, 145.8) with ten positive samples (Fig 6A). Similar trends were observed with RBD antibodies where we observed an approximate 3-fold reduction in the GMT of RBD antibodies from 266.0 (95% CI: 120.2, 368.1) in June 2021 to 76.5 (95% CI: 36.7, 159.5) in Dec 2021/Jan2022. The samples from Omicron wave showed a spike in RBD antibody titers which increased to 935.3 (95% CI: 440.4, 1986) (Fig 6B). To establish virus neutralization titer assays for the Omicron variant, we first performed conventional plaque assays with the virus stocks using both Calu-3 and Vero E6 cells and found delayed and small plaque formation in Calu-3 cells as compared to the Delta variant (S5A Fig). However, unlike the Delta variant, the Omicron variant did not produce plaques up to 48 h post-infection in Vero E6 cells. As Calu-3 cells are slow-growing and plaque assays are not amenable for high-throughput neutralization assays, we first tested the ability of the Omicron variant to form infectious foci by antibody staining with anti-spike and anti-nucleocapsid antibodies in Vero E6 cells. While the Delta variant formed clear discernable infectious foci with both the antibodies, the same was less distinct with anti-spike antibodies for the Omicron variant although it was countable by the spot reader (S5B Fig). Staining with nucleocapsid antibodies produced better foci in the case of Omicron variant but the quality of foci was not comparable with the Delta variant (S5B Fig). We next tested secondary antibodies conjugated with fluorescent dye instead of horseradish peroxidase enzyme (HRP) and visualized the infectious foci in fluorescence mode. Use of fluorescence-based detection was far superior to HRP-based enzyme-substrate detection for the Omicron variant (S5C Fig). The assay showed consistency in performance as measured by the antibody titers of an in-house pooled convalescent reference serum (S5D Fig). We adopted this method for comparing the neutralizing antibody titers against the Delta and Omicron variants. The neutralizing antibody titers for the Delta variant, as measured by FRNT assay, also showed about three-fold reduction from a GMT of 257.5 (95% CI: 145.8, 454.7) in June 2021 to 87.5 (49.7, 154.1) in Dec 2021/Jan2022 (Fig 6C). However, as expected from the ELISA titers, the GMT of neutralizing antibodies increased significantly to 914.0 (95% CI: 468.7, 1782) in samples from the third wave collected in Feb 2022 indicating re-infection in most of these individuals. As shown by us and others [14,16], the neutralizing antibody titers for Omicron variant was drastically reduced at all the time points relative to that of Delta variant (Fig 6D). Nine out of 15 samples had FRNT_50_ value above the level of detection after vaccination in June 2021. The GMT of neutralizing antibodies for the Omicron variant was 26.9 (95% CI: 15.6, 46.4) in these samples. The number of samples positive for Omicron antibodies reduced to six out of 15 bringing down the GMT of neutralizing antibodies to 18.3 (95% CI: 11.6, 28.7) by Dec 2021/Jan2022. As expected, the surge in Omicron infections in the study area was reflected in the GMT of neutralizing antibodies in samples from Feb 2022 (Fig 6D). Surprisingly, the samples from Omicron surge showed relatively lower titer, a GMT of 261.9 (95% CI: 113.0, 607.5) for BA.1 as compared to a GMT of 914.0 (95% CI: 468.7, 1782) for the Delta variant suggesting that natural infection with Omicron variant generates significantly higher levels of antibodies to the Delta variant.

As BA.2.75 sub-lineage of the Omicron variant emerged and became the predominant variant circulating in India since July 2022, it was reported that the antibodies from vaccinated and/or previously-infected individuals showed the most drastic reduction in their ability to neutralize BA.2.75 [43]. We had enrolled participants (n = 20) who were either COVID-19 RT-PCR positive in the BA.1/BA.2 surge or had symptomatic respiratory infection but got no COVID-19 testing done during the months of Jan/Feb 2022, before the emergence of BA.2.75 in India. These participants were confirmed to be recently infected based on increase in antibody titers in RBD or N-ELISA compared to the previous bleed collected in Jan 2022. The GMT of neutralizing antibodies for BA.1, BA.2 and BA.2.75 sub-lineages in these samples were 502.2 (95% CI: 316.6, 796.4), 822.1 (95% CI: 530.9, 1273) and 276.0 (95% CI:154.6, 492.6) (Fig 6E) which was around one order of magnitude more than the NT50 values obtained for samples from individuals (n = 4) who remained unexposed to the Omicron variants (Fig 6F). These results confirm previous observations that BA.2.75 VoC has the greatest ability to escape neutralization from breakthrough infections of Omicron sub-lineages [43]. As we recently reported minimal to no neutralizing antibodies against the Omicron variant in subjects with vaccine-induced or hybrid immunity at the beginning of the surge in COVID-19 cases in December 2021 [16] and none of the participants had received a booster dose of vaccine, our data suggests that enhanced neutralizing antibody titers observed in this study in the cohort of individuals (Fig 6E) is due to natural exposure to SARS-CoV-2 Omicron variants. The highest titers of neutralizing antibodies elicited against the Delta variant in breakthrough infections of Omicron lineages further confirms the hypothesis of original antigenic sin proposed by others which may explain the lowest level of neutralizing antibody titers observed against BA.2.75 [44]. We were able to collect a six-month follow-up sample from nine of the participants who were infected with one of the Omicron variants prior to Feb 2022 (Fig 6G–6J) As expected neutralization titers dropped significantly for the Delta (Fig 6G), BA.1 (Fig 6H) and BA.2.75 (Fig 6I) variants but not for BA.2 (Fig 6J) as few of the participants showed an increase in titers for BA.2 most likely due to natural infection with BA.2 or one of the BA.2 sub-lineages during this period. Nevertheless, the relative antibody titers remained the highest for the Delta VoC and lowest for the BA.2.75 VoC.

### Copper and Iron (II) salts inhibit both Delta and Omicron variant infection

**Fig 7:**
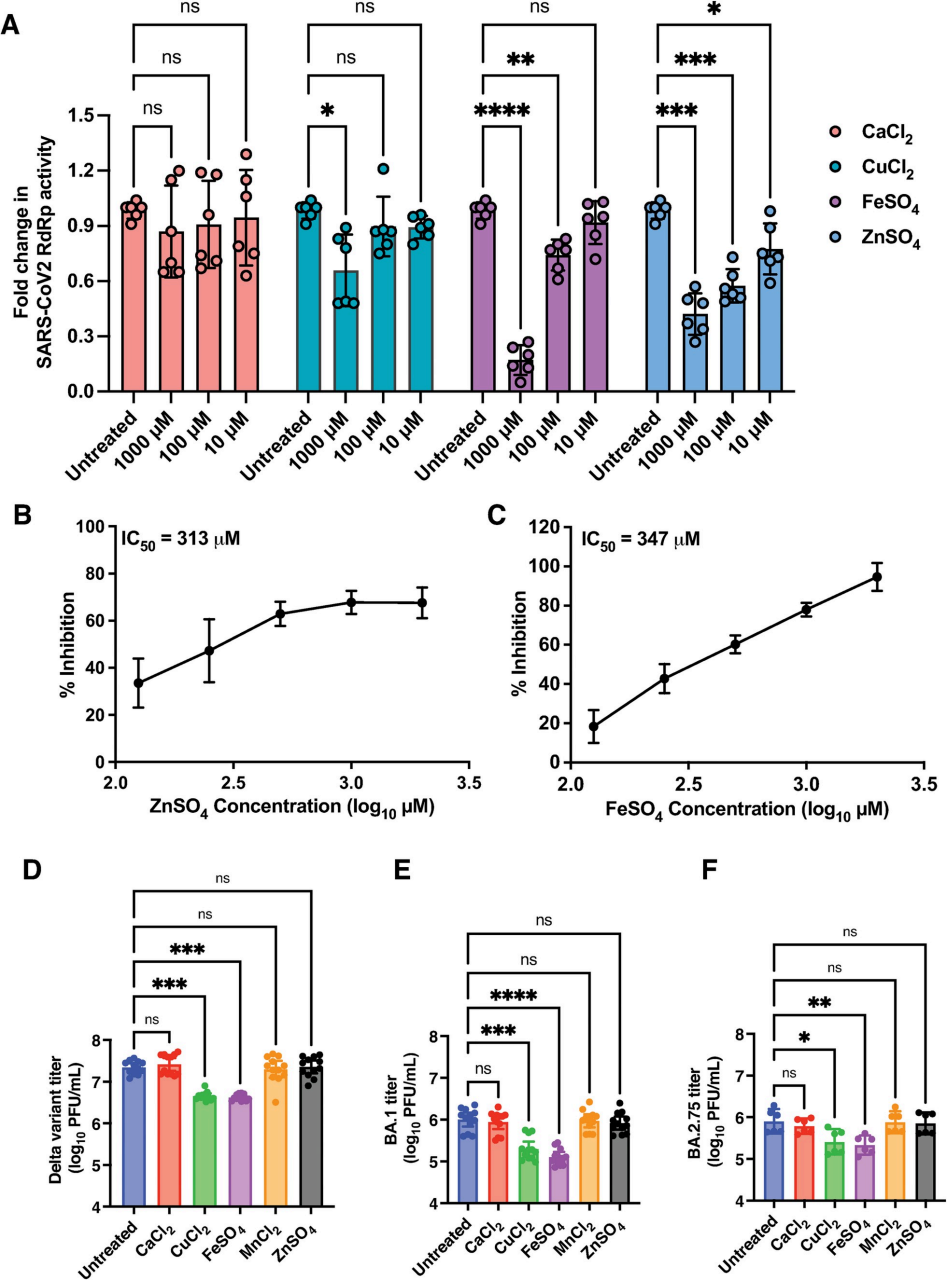
Iron and copper salts significantly inhibit SARS-CoV-2 infection. (A) SARS-CoV-2 RdRp activity assay was performed in the presence of indicated concentrations of CaCl_2_/CuCl_2_/FeSO_4_/ZnSO_4_ in the standard reaction mix containing MgCl_2_ and MnCl_2_ and the RdRp complex (nsp7-nsp8-nsp12) of SARS-CoV-2. (B) RdRp assays were performed in the presence of varying of concentrations of ZnSO_4_ or (C) FeSO_4_ to determine the IC_50_ values. To determine the effect of metal ions in infection, Calu-3 cells were infected with indicated variants at 0.3 MOI and media containing 50 μM each of the indicated salts were added after virus adsorption. At 24 h pi, supernatants were collected and viral titers were measured by plaque assay. The graph indicates the virus titers of (D) Delta (E) BA.1 and (F) BA.2.75 variants. Data are from two or three independent experiments. Error bars represent geometric mean with 95% CI. Statistical significance was estimated by Kruskal-Wallis test with Dunn’s multiple comparison test. ns: non-significant, * P<0.05, ** P<0.01, *** P<0.001, **** P<0.0001.

Divalent cations play an important role in the replication of RNA viruses [45] and previous reports have suggested inhibition of coronavirus replication by Zn salts [46]. We established the fluorescence-based SARS-CoV-2 RNA-dependent RNA polymerase (RdRp) assay using purified nsp7, nsp8 and nsp12 proteins with minor modifications based on previous reports [47–49]. RdRp assays were performed in the presence of 10 μM, 100 μM or 1000 μM of CaCl_2_/CuCl_2_/FeSO_4_/ZnSO_4_. CaCl_2_ did not affect RdRp activity under these conditions. At 1000 μM, CuCl_2_ showed about 35% reduction in RdRp activity while FeSO_4_ and ZnSO_4_ showed 85% and 60% inhibition respectively at the same concentration (Fig 7A). At 100 μM, FeSO_4_ showed 25% reduction in activity which was significant compared to untreated while ZnSO_4_ showed 52% inhibition at this concentration (Fig 7A). Only ZnSO_4_ was capable of inhibiting SARS-CoV-2 RdRp activity albeit by only 22% at 10 μM (Fig 7A). The 50% inhibitory concentration (IC_50_) of ZnSO_4_ and FeSO_4_ in RdRp assays was 313 μM and 347 μM respectively (Fig 7B and 7C). To further confirm the validity of these *in vitro* inhibition in cell culture, we infected Calu-3 cells with either the Delta variant or the BA.1 or BA.2.75 sub-lineages of the Omicron variant and growth medium supplemented with 50 μM each of CaCl_2_/CuCl_2_/FeSO_4_/MnCl_2_/ZnSO_4_ was added after virus adsorption and cells were cultured for 24 h. This concentration of salts was shown to not affect viability of the cells (S6 Fig). Virus titers in the infected culture supernatants were estimated by plaque assays. Contrary to RdRp assay results, ZnSO_4_ at 50 μM concentrations failed to show any inhibition of either the Delta or the Omicron variants in Calu-3 cells (Fig 7D–7F). Similarly, neither CaCl_2_ nor MnCl_2_ had any impact on virus titers. However, both CuCl_2_ and FeSO_4_ showed significant inhibition of virus titers of both Delta and the BA.1 and BA.2.75. (Fig 7D–7F). Therefore, our results suggest that copper and iron salts may have both direct and indirect antiviral activity against SARS-CoV-2 when added post-infection.

## Discussion

SARS-CoV-2 Delta variant was shown to be more infectious with a higher reproduction number compared to the ancestral Wuhan strain and the duration from PCR positivity or symptoms with this variant was shorter as compared to other SARS-CoV-2 strains leading to faster transmission and severe disease [50]. The 13 additional mutations in the spike protein of delta variant compared with the ancestral virus conferred a combination of advantages namely, neutralization escape by antibodies generated from either vaccination or prior infection, higher replication fitness relative to ancestral virus or the alpha variant, the P681R mutation enabling efficient cleavage of precursor spike to active S1/S2 and higher cell-cell fusion and syncytium formation and more efficient entry [16,32,33,51], Using a a hierarchical Bayesian regression model to analyze 6.4 million SARS-CoV-2 genome sequences available in the GISAID database, a recent study identified the BA.1 and BA.2 sub-lineages to be the fittest viruses which have evolved over the course of the pandemic [52], In addition to mutations in the spike protein, a number of non-spike mutations were also predicted to play a role in replication fitness [52]. In our study, the Delta variant showed enhanced disruption of epithelial barriers and junction proteins suggesting that the enhanced cytopathic effect and syncytia formation combined with the ability to escape from neutralizing antibodies has provided a growth advantage for Delta variant although the exact mechanism behind enhanced virulence and pathogenesis remains to be deciphered [1–3,53]. Contrary to the enhanced pathogenicity of the Delta variant, the Omicron variant and its sub-lineages BA.1, BA.2 and BA.2.75 showed slower replication kinetics at early stages but caught up with delta variant between 24 to 36 h pi. The three sub-lineages of Omicron variant had milder effect on epithelial barrier functions which correlates with less number of severe cases, hospitalization and lower number of deaths due to of SARS-CoV-2 infections in India since the emergence of Omicron and its sub-variants which is similar to what has been reported from other countries [54]. We showed that BA.2.75 variant had similar entry and replication kinetics compared to BA.2 in polarized air-liquid culture models as reported by others [25]. The clinical samples for this study was collected during the second and third wave of COVID-19 in India during which the Delta and Omicron (BA.1 and BA.2) variants were the predominant circulating virus strains. Cohort data of patients with mild COVID-19 infection has shown that RBD antibodies decay with a half-life of 69 days [55]. We observed a three-fold reduction in RBD antibody and neutralizing antibody titers after six months. However, most of the samples had no neutralizing antibodies against the Omicron variant after six months of vaccination suggesting that waning neutralizing titers are an important determinant of reinfections and may also contribute to increased susceptibility to new variants of concern that escape pre-existing humoral immunity. The high-throughput neutralizing antibody assay we established in this study enabled us to circumvent the challenges of estimating neutralizing antibody titers for the Omicron variant as it grew poorly and did not form distinct plaques or infectious foci like the Delta variant. Currently, BA.1 and BA.2 sub-lineages of the Omicron variant are circulating in India and reports have raised concern about the enhanced pathogenicity or transmissibility of BA.2 sub-lineage due to unique mutations in the spike region [42,56]. We show that both BA.1 and BA.2 sub-lineages of Omicron variant have comparable replication kinetics in Calu-3 cells. Our sequencing data suggests that the BA.2 sub-lineage of the Omicron variant was the predominant circulating virus at the time of the study and relatively higher levels of neutralizing antibodies to BA.2 was observed in study participants as compared to BA.1 further confirming that BA.2 may have been the infecting virus. We show that participants who were naturally infected with the Omicron variant generated neutralizing antibodies against both BA.1 and BA.2 sub-lineages indicating that exposure to any of the sub-lineages of Omicron would confer cross-protection against other members of the sub-lineage in agreement with other reports [20,57]. Interestingly, higher titers of neutralizing antibodies were observed against the Delta variant which was no longer in circulation indicating a broader cross-protective immune response most likely due to conserved B-cell and T-cell epitopes in the Omicron variant that could have triggered the memory responses in people with hybrid immunity [58,59]. Thus, natural infection with Omicron variant may have acted as a booster eliciting cross-reacting antibodies across some of the Omicron sub-lineages and other VoCs as reported by others previously [60,61]. The BA.2.75 sub-lineage which became the predominant sub-lineage circulating in India while this manuscript was under review, was shown to enter cells more efficiently compared to the Delta variant and initiate viral replication faster. BA.2.75 was shown to be more fusogenic in cell culture models and more pathogenic in hamster models [62,63]. However, in our cell culture models, both the BA.2 and BA.2.75 lineage viruses were compromised in their ability to disrupt epithelial barriers. Although BA.2.75 infections peaked in the months of July and August in India, there were no reports of any substantial increase in hospitalizations or deaths in India due to COVID-19 infections. Therefore, we propose that although the newer sub-lineages of Omicron are evolving with more efficient cellular entry capabilities, faster replication and are evading humoral immunity from prior infection or vaccination, these newer variants also have compromised ability to cause severe disease. Repeated exposure to newer variants of SARS-CoV-2 omicron lineages may elicit recall responses and ensure maintenance of protective levels of neutralizing antibodies and T-cell responses to prevent severe disease [61].

Several studies have reported that interferons expressed in the lower respiratory tract contribute to tissue damage, impair regeneration of lung epithelium and contribute to morbidity observed in COVID-19 patients [64,65]. We observed similar levels of induction of *IFN-β* and *IFN-λ1* and the downstream effectors namely *ISG15* and *OAS1* in cells infected with the Delta variant and with the ancestral B.6 lineage virus suggesting that suppression of innate immune responses is not a contributory factor in increased pathogenesis observed with the Delta variant. Despite lower levels of infection, the Omicron (BA.1) variant induced higher levels of interferon pathway genes. Since the Omicron variant was shown to mostly replicate in the upper respiratory tract [66,67], this suggests that interferon-dependent antiviral responses may limit viral replication and further tissue spread thus resulting in milder symptoms and resolution of infection. Interestingly, the BA.4 and BA.5 variants that emerged in mid 2022 were shown to suppress interferon induction [68] although these variants did not have any major impact on the case load or clinical outcomes in India. Therefore, interferon therapy targeted towards the upper respiratory tract may be more effective in the case of infection with the Omicron variants. In addition to interferons, our results suggest that supplementation with copper and iron salts may also augment the antiviral strategy for SARS-CoV-2 as both CuCl_2_ and FeSO_4_ inhibited both the Delta and Omicron variants in Calu-3 cells.

Vaccines have played a major role in bringing the COVID-19 pandemic under control. However frequent emergence of SARS-CoV-2 variants requires adjunct approaches to deal with infections. Micronutrient supplementation could be an effective and safe strategy. Ferric ammonium citrate (FAC) was shown to inhibit a number of RNA viruses including Influenza A virus, Zika virus and Hepatitis C virus in both cell culture and animal models and it was shown that treatment with FAC perturbed endosomal release of the internalized virus in the case of Influenza A virus [69]. SARS-CoV-2 nsp12 has been shown to bind Fe and Iron-Sulfur cluster binding was required for optimal activity of the RdRp [70]. However, there have been no direct demonstration of the effect of Fe salts on either the RdRp activity *in vitro* or in lung epithelial cells. We show here that FeSO_4_ inhibited both RdRp activity and infection in Calu-3 cells. The inhibitory concentration in cell culture was much below the *in vitro* IC_50_ concentrations. Contrary to our findings, iron overload is considered as a risk factor for severe COVID-19 disease and iron chelation has been proposed as a treatment option [71,72]. Therefore, the exact mechanism of action behind inhibition of SARS-CoV-2 infection by FeSO_4_ warrants further investigation. Interestingly, CuCl_2_ which inhibited RdRp activity at 1 mM also inhibited virus infection in Calu-3 cells at 20 times below this concentration indicating that both Fe an Cu ions may act on SARS-CoV-2 both directly and via host pathways. Previous reports have shown that copper-coated surfaces can inactivate SARS-CoV-2[73] and other enveloped and non-enveloped viruses are susceptible to copper ions either due to direct effect of copper ions on viral particles or viral genome or due to induction of reactive oxygen species. Therefore, copper supplementation during viremic phase may further augment antiviral strategies for COVID-19 [74–76]. Zinc acetate, in combination with zinc ionophore pyrithione, was shown to inhibit SARS-CoV-1 infection in Vero E6 cells and zinc ions was shown to directly inhibit RdRp activity of SARS-CoV-1[46]. SARS-CoV-2 proteome encodes for a number of metalloproteins including zinc-binding proteins such as nsp2, nsp9, nsp10/16 complex, nsp12 and proofreading exoribonuclease nsp14 (Please see Uniprot ID: P0DTD1 · R1AB_SARS2). Some of the critical aspects of viral replication such as RdRp activity, 2’-*O* methylation, capping depends on intracellular zinc [77,78]. Zinc ejection from some of these enzymes has been proposed as an attractive antiviral target [79]. However, despite ZnSO_4_ inhibiting the activity of SARS-CoV-2 RdRp *in vitro*, we saw no effect on SARS-CoV-2 Delta and Omicron variants at 50 μM concentration. Whether higher concentrations of zinc salts are required to achieve significant inhibition of these variants in cell culture needs further investigation.

## Methods

List of reagents and resources used in this manuscript is provided in S1 Table.

### Ethics statement

The study was approved by the Institutional Ethics Committee for human research at Employees State Insurance Corporation Medical College and Hospital (No.134/R/10/IEC/22/2021/02) and Translational Health Science and Technology Institute (THS 1.8.1/ (93)). Written informed consent was obtained from all the participants.

### Human samples

ChAdOx1 nCoV-19 cohort: Subjects (age 25–46) visiting ESIC Medical College & Hospital, Faridabad for vaccination were enrolled in the study after obtaining written informed consent. About 4 ml of whole blood was collected for serum preparation four weeks after the first and second dose of vaccination and a follow-up sample was collected after six months. Nasopharyngeal/Oropharyngeal (NP/OP) swabs were collected from patients with symptoms of COVID-19 infection. Total RNA was isolated to detect SARS-CoV-2 using COVIDsure multiplex real-time RT-PCR kit (Trivitron Healthcare) either at Employees State Insurance Corporation (ESIC) Medical College & Hospital or at the bioassay laboratory Translational Health Science and Technology Institute, Faridabad. Clinical presentations were mild to moderate fever, dry cough, and loss of sense of smell and taste. All COVID-19 positive patients were self-isolated and recovered without any need for clinical intervention or hospitalization. A follow-up blood sample was collected after 3–4 weeks post-recovery in both second (May 2021) and third wave (Feb 2022).

### Cells and viruses

Calu-3 cells and Vero E6 cells were procured from American Type Culture Collection (ATCC) and European Collection of Authenticated Cell Cultures (ECACC) respectively. Both cell lines were maintained in Dulbecco’s Modified Eagle Medium (DMEM) (HiMedia) supplemented with 10% fetal bovine serum (FBS), 100 units/ml of Penicillin- Streptomycin-Glutamine (PSG), and 1X non-essential amino acid (NEAA). In addition to this, 25 mM HEPES was added to the Vero E6 culture medium. Different variants of SARS-CoV-2 i.e., ancestral (B.6, Genbank: MW422884.1), kappa (B.1.617.1, Genbank: MZ356902.1), delta (B.1.617.2, Genbank: MZ356566.1), and omicron (BA.1 GISAID: EPI_ISL_6716902; BA.2 GISAID ID: EPI_ISL_10638432 and BA.2.75 GISAID: EPI_ISL_14507883 sub-lineages) were propagated in Calu-3 cells and passaging was limited to five passages.

### Growth curve experiments

All experiments with infectious SARS-CoV-2 virus were performed at the infectious disease research facility, which is a biosafety level-3 laboratory. Calu-3 cells were seeded in 48-well or 24-well plates and infected with indicated SARS-CoV-2 virus isolates two days post-seeding. Infected culture supernatants were collected at indicated time points to estimate viral titers by plaque assay on Vero E6 cells. Total RNA was extracted from cells collected at respective time points and viral copy numbers (N gene) were estimated by quantitative RT-PCR using RNAse P gene for normalization as described previously [53].

### Plaque assay

Vero E6 cells were used for virus titration by plaque assay method. The supernatants were collected from infected cells at indicated time points. The supernatants were ten-fold diluted using growth medium with 2% FBS. After 1h of viral adsorption, virus inoculum was removed. Overlay medium with 0.5% carboxymethylcellulose (CMC) was added to the cells. The plates were incubated at 37° C for 48 h for all SARS-CoV-2 variants except omicron where the incubation period was 72 h. After the incubation period, cells were fixed with 3.7% formaldehyde solution followed by incubation for 10 min at room temperature (RT). Cells were stained with crystal violet solution and plaques were observed.

### Trans-epithelial electrical resistance (TEER) and confocal microscopy

Calu-3 cells were seeded at 30,000 on 3 μm pore size transwell inserts (Corning—3415) and grown for 21 days. TEER was monitored on alternate days using a chopstick electrode (Millipore) and the medium was changed on the same day. On day 22 post-seeding, cells were infected with indicated variants of SARS-CoV-2 at 0.3 MOI. After 1 h of viral adsorption, virus inoculum was removed and cells were washed twice with 1X PBS. Growth medium with 10% FBS was added to the cells. TEER was monitored at indicated time points. At 48 h pi, culture inserts were washed with cold 1X PBS and incubated with cold methanol at −20°C for 20 minutes. Cells were further incubated with IMF buffer (20 mM HEPES, pH 7.5, 0.1% Triton X-100, 150 mM sodium chloride, 5 mM EDTA and 0.02% sodium azide as a preservative) for 5 min at room temperature (RT) and all further washes were performed with IMF buffer. Non-specific antibody binding sites were blocked by incubating cells with IMF buffer containing 2% normal goat serum for 10 min at RT. Membranes were cut out using a scalpel blade and transferred to a 48-well plate. Cells were washed three times followed by incubation with antibodies against SARS-CoV-2 nucleocapsid, occludin, and β-catenin in IMF buffer for 1 h at RT. Cells were washed followed by incubation with secondary antibodies tagged with Alexa Fluor 488/568/633 (Molecular probes) for 30 min at RT by avoiding exposure to light. Cells were washed with IMF buffer three times and stained with DAPI at 1:10,000 dilution for 10 min. Cells were washed with PBS, mounted on glass slide using antifade solution (Molecular probes), and images were captured at 100X magnification using FV3000 confocal microscope (Olympus). Acquired images were processed using CellSens software (Olympus) and projections of Z-stack (maximum intensity) are shown in figures.

### Focus-forming units (FFU) assay

The supernatants were collected from infected cells at indicated time points. The supernatants were ten-fold diluted using growth medium with 2% FBS. After 1h of viral adsorption, virus inoculum was removed. Overlay medium with 1.5% carboxymethylcellulose (CMC) for all SARS-CoV-2 variants except for Omicron where 1% CMC was used. The plates were incubated at 37° C for 28 h for omicron and 24 h for all other SARS-CoV-2 variants. After incubation time, cells were fixed with formaldehyde solution followed by permeabilization with IMF buffer for 20 min incubation. Further, cells were stained with anti-spike RBD rabbit polyclonal antibody dilution at a dilution of 1:2000 for 1 h, followed by incubation with secondary antibody i.e., Alexa flour 488-conjugated anti-rabbit antibody at 1:500 dilution for 1 h. For omicron isolate, anti-nucleocapsid primary antibody was used at a dilution of 1:2000. This was followed by incubation with secondary antibody i.e., Alexa flour 488-conjugated goat anti-mouse IgG secondary antibody at 1:500 dilution. Fluorescent foci indicating infected cells were observed and counted using AIDiSpot reader using FITC channel.

### Western blot

At indicated time points, cells were washed twice with cold PBS on ice and were collected in 200 μl of 1X Laemmli buffer with protease inhibitor mix and 1 mM Phenylmethylsulfonyl fluoride. Lysates were incubated on ice for 10 min and sonicated (amplitude: 30%, time: 5 sec ON/10 sec OFF, total cycles: 10) on ice. Cell lysates were centrifuged at 13,000 x *g* for 15 min at 4°C. 5 μl of each sample was resolved on 12% SDS-PAGE. Gels were transferred onto polyvinylidene difluoride membrane for 2 h and the SARS-CoV-2 infection was analyzed by probing the blot with the SARS-CoV-2 nucleocapsid antibody. GAPDH antibody was used as a loading control. Primary antibody incubation was followed by HRP-conjugated secondary antibodies. *Luminol*-*based chemiluminescent substrate was added to the blots and s*ignals were detected using a gel documentation system (Azure biosystems C400).

### Virus entry experiment

Calu-3 cells were infected with the above-indicated variants of SARS-CoV-2 at 5 MOI. After 1 h of viral adsorption, the virus inoculum was removed and cells were washed with 1X PBS. Cells were trypsinized and collected in complete growth medium and centrifuged at 200 x *g* for 5 min. The supernatants were discarded and the cell pellets were washed twice with 1X PBS followed by centrifugation. In the final step, the cell pellets were collected in 350 μl lysis buffer. Viral copy numbers were estimated as described in the above section.

### Quantitative RT-PCR assay

Calu-3 cells were infected with ancestral, delta, and omicron variants of SARS-CoV-2 at a MOI of 0.3. At indicated time points, the supernatant was collected for estimating viral titres by plaque assay, and cells were collected in lysis buffer provided in the RNA isolation kit as described in the above section. For interferon pathway, 500 ng RNA was used to erase genomic DNA and reverse transcribed using PrimeScript RT reagent kit with gDNA eraser kit. The indicated genes were quantitated by SYBR green chemistry and *GAPDH* was used as housekeeping control gene for normalization. The list of primers used is provided in the key resources table. Data were analyzed using the *ΔΔC*_*T*_ method, where *C*_*T*_ is cycle threshold.

### Air-liquid interface model and confocal microscopy

Calu-3 cells were seeded at a density of 30,000 cells in 3 μm pore size polycarbonate membranes in 24 well-plate with corresponding 200 μl culture medium on the apical side and 800 μl culture medium was added into the basolateral chamber. Cells were left to grow submerged for 8 days. The culture medium was changed on alternate days and TEER values were monitored. On day 9, the medium was removed from the apical chamber to obtain air-liquid interface (ALI) conditions. Cells were cultured in ALI conditions for 20 days. After obtaining stable TEER values, on day 21, cells were washed with 1X Hanks’ balanced salt solution (HBSS). Cells were infected with either the Delta or the Omicron variants of SARS-CoV-2 at 0.3 MOI. After 1 h of viral adsorption, cells were washed twice with HBSS, and culture medium was added to the basolateral side. To assess the effect of SARS-CoV-2 on epithelial cells barrier, TEER was monitored at indicated time points. Supernatants were collected from basolateral surface for measuring viral titres. At 36 h pi, cells were washed with cold PBS and fixed in ice-cold methanol for 20 min at −20°C. The cells were stained against junction proteins along with nucleocapsid antibodies as described above in the confocal microscopy section.

### Quantitative Nucleoprotein (N) and RBD ELISA

ELISA for nucleocapsid (N) and recombinant spike protein receptor-binding domain (RBD) was performed as described earlier. Detailed method description is provided in previous reports [19,80].

### Virus microneutralization assay

Virus microneutralization assay by focus reduction neutralization titer (FRNT) assay using indicating virus isolates was performed as described earlier [19,80].

### RNA-dependent RNA polymerase (RdRp) activity assay

The Quant-iT PicoGreen dsDNA reagent was used to establish a fluorophore-based RdRp activity system. Adenosine 5′-triphosphate (ATP) was purchased from Sigma–Aldrich. RdRp RNA template with self-hairpin primer used for RdRp assays in this study as following 5′-UUUUUUUUUUUUUUUUUUUUUUUUUUUUUUAACAGGUUCUAGAACCUGUU-3′ (Eurofins). Each reaction (final volume 30 μl) was carried out in 96 well black flat-bottom plates in triplicates. 150 nM of nsp12 and 1.5 μM of nsp7 & 8 was mixed in a reaction buffer (50 mM HEPES pH 8, 5 mM DTT, 10 mM KCl, 2 mM MgCl_2_, 2 mM MnCl_2_) to make up to 15 μl. Indicated concentrations of ZnSO_4_, CaCl_2_, CuCl_2,_ and 5 μl of 200 nM poly-U were added to the reaction buffer. The mixture was incubated at 30°C for 5 min. The reactions were initiated by adding 5 μl of 500 μM ATP to the reaction mixture. The plate was incubated at 30°C for 30 min. The reaction was stopped by adding 20 μl EDTA (100 mM). The Quant-iT PicoGreen dsDNA reagent was diluted to 1/800 in 1X TE buffer (10 mM Tris-HCl, 1 mM EDTA, pH 7.5) and 50 μl of this reagent was added to each well of the plate. The plate was incubated in the dark for 30 min at RT. The fluorescence signal was assessed using microplate reader at excitation of 485 nm and emission of 582 nm.

### Inhibition experiments

Calu-3 cells were seeded at a density of 90,000 per well in 48 well-plate. Cells were infected with indicated SARS-CoV2 variants at 0.3 MOI. After viral adsorption, cells were washed twice with 1X PBS. CaCl_2_, CuCl_2_, FeSO_4_, MnCl_2,_ and ZnSO_4_ were added each in growth medium in serum-free condition to a final concentration of 50 μM. After 24 h pi, supernatants were collected and viral titers were measured by plaque assay.

### Statistical tests

Data were analysed and charts were prepared using GraphPad Prism software. All experiments were performed with two or more replicates and graphs have been prepared representing data from at least two independent experiments. Figure legends indicate error bars and statistical tests conducted for estimating P values.

## Supporting information

S1 Table List of Reagents and Resources used in the study. (DOCX)

S1 Methods A separate file has been uploaded for description of methods for Whole genome sequencing of SARS-CoV-2 using Nanopore platform, Nanopore Analysis Method, Cell viability assay. (DOCX)

S1 Data Source data containing the numerical values used in generation of Figs 1–7. (XLSX)

S2 Data Source data containing the numerical values used in generation of S1–S6 Figs. (XLSX)

S1 Fig COVID-19 diagnostic testing. (A) NP/OP samples were tested by RT-PCR to diagnose COVID-19 infection and % positivity (i) and number of positive cases relative to the total number of samples tested (ii) from April 2020 to July 2021 is shown. (B) Threshold cycle (Ct) values of RNA samples tested for COVID-19 during the same period is shown and the values are segregated into four categories to indicate very high (< 20 Ct), high (20–25 Ct), moderate (25–30 Ct) and low (>30 Ct) viral burden in the first sample collected for diagnosis.
(TIF)

S2 Fig Omicron (BA.1) variant has milder effect on epithelial junctions. Calu-3 cells were grown on transwell inserts under air-liquid interface (ALI) conditions. Cells were infected with Delta and Omicron variants at 0.3 MOI. At 36 h pi, cells were fixed and stained with SARS-CoV-2 nucleocapsid antibody followed by Alexa Fluor 488-conjugated secondary antibodies for visualization. Nuclei were stained with DAPI. Images were captured at 20X magnification. Images were analyzed using cellSens software and Z-projection images with maximum intensity are shown in the figure. Scale bar is 50 μM. (B) Graph indicates TEER values relative to mock infection after infection at indicated time points from two independent experiments. (Mean and error with range). Statistical significance was estimated by two-way ANOVA with Tukey’s multiple comparisons test. (C) Viral titers were measured in supernatants by focus-forming units. Error bars represent (Mean ± SD) (D) At 36 h pi, cells were fixed and stained with occludin, β-catenin and SARS-CoV-2 nucleocapsid antibody followed by Alexa Fluor dye-conjugated secondary antibodies for visualization. Nuclei were stained with DAPI. Images were captured at 100X magnification. Images were analyzed using cellSens software and Z-projection images with maximum intensity are shown in the figure. Scale bar is 10 μM. ns: non-significant, **** P<0.0001.
(TIF)

S3 Fig Distribution of SARS-CoV-2 lineages in National Capital Region of India between the months of September 2021 to January 2022. Whole genome sequencing of COVID-19 positive diagnostic samples for the indicated period. B.1.617.2 (Delta); AY.* (Delta plus); Omicron lineages (B.1.529, BA.1 and BA.2).
(TIF)

S4 Fig Distribution of SARS-CoV-2 lineages in India. Virus circulation in the months of (A) July and (B) August 2022 as per the sequences deposited in GISAID database.
(TIF)

S5 Fig Growth characteristics of Omicron variant and establishment of FRNT assay. (A) Calu-3 or Vero E6 cells were incubated with 10-fold serial dilution of Omicron (BA.1) variant to determine virus titers by plaque assay. Plates were fixed at 24 and 48 h pi and stained with crystal violet. (B) Vero E6 cells were infected with a pre-determined dilution of Omicron variant for focus-forming unit assay using anti-spike and anti-nucleocapsid antibodies followed by HRP-conjugated secondary antibody. Foci were developed using TrueBlue substrate. (C) Vero E6 cells were infected with a pre-determined dilution of Omicron variant for focus-forming unit assay using anti-spike and anti-nucleocapsid antibodies followed by Alexa488-conjugated secondary antibody. Foci were visualized under fluorescence channel in the reader. (D) FRNT assay using fluorescence method to determine the neutralization titers of antibodies against the Delta and Omicron variants. 50% neutralization titer of antibodies (NT_50_) is given from at least five experimental replicates (Mean ± SD).
(TIF)

S6 Fig Effect of divalent cations on cell viability. Calu-3 cells were treated with indicated salts at a concentration of 50 μM for 24 h. Cell viability assay was performed using CellTiter-Glo luminescent cell viability assay. Data from two experiments are presented as Mean + SD.
(TIF)
