# Supplementary material for: BA.1, BA.2 and BA.2.75 variants show comparable replication kinetics, reduced impact on epithelial barrier and elicit cross-neutralizing antibodies
Source: PLoS Pathog. 2023 Feb 24;19(2):e1011196. doi: 10.1371/journal.ppat.1011196 (PMC9994724; doi:10.1371/journal.ppat.1011196)
Supplement: S1 Table — (DOCX) [file ppat.1011196.s001.docx]

**Supporting Table 1: Reagents and Resources used in the study**

| **REAGENTS OR RESOURCES** | **SOURCE** | **IDENTIFIER** |
| --- | --- | --- |
| **Antibodies** |  |  |
| SARS-CoV-2 Nucleocapsid | GenScript | Catalog no. A02048 |
| SARS-CoV-2 Spike RBD | Sino Biological | Catalog no. 40592-T62 |
| β-catenin | BD Biosciences | Catalog no. 610154 |
| Occludin | Invitrogen | Catalog no. 71-1500 |
| GAPDH | Cell Signaling Technology | Catalog no. 2118S |
| Alexa fluor 488 goat anti-human IgG | Invitrogen | Catalog no. A-11013 |
| Alexa fluor 488 goat anti-mouse IgG2b | Invitrogen | Catalog no. A-21141 |
| Alexa fluor 633 goat anti-mouse IgG1 | Invitrogen | Catalog no. A-21126 |
| Alexa fluor 568 donkey anti-rabbit IgG | Invitrogen | Catalog no. A-10042 |
| Goat Anti-Rabbit lgG (H+L) Horseradish Peroxidase conjugate | Invitrogen | Catalog no. G-21234 |
| Goat anti-Mouse IgG (H+L) Cross-Adsorbed Secondary Antibody, HRP | Invitrogen | Catalog no. A16072 |
| HRP-Goat Anti- Human IgG | Jackson ImmunoResearch | Catalog no. 109-035-170 |
|  |  |  |
| **Virus strains** |  |  |
| BL-THSTI 2010D (B.6) | GenBank accession:  MW422884.1 |  |
| Kappa (B.1.617.1) | GenBank accession: MZ356902.1 |  |
| Delta (B.1.617.2) | GenBank accession: MZ356566.1 |  |
| Omicron BA.1 | GISAID-accession no EPI_ISL_6716902 |  |
| Omicron BA.2 | GISAID-accession no EPI_ISL_10638432 |  |
| Omicron BA.2.75 | GISAID-accession no EPI_ISL_14507883 |  |
|  |  |  |
| **Media and chemicals** |  |  |
| Dulbecco’s Modified Eagle Medium (DMEM), High glucose | HiMedia | Catalog no. AL007A |
| Minimum Essential Media (MEM) | Thermo Fisher/Gibco | Catalog no. 11090073 |
| Penicillin-Streptomycin-Glutamine (PSG) 100X | Thermo Fisher | Catalog no. 10378016 |
| Antibiotic solution 100X liquid | HiMedia | Catalog no. A001 |
| Non-Essential Amino Acids Solution (100X) | Thermo Fisher | Catalog no. 11140-050 |
| Fetal Bovine Serum (FBS) | Thermo Fisher | Catalog no. 16140-071 |
| Trypsin-EDTA (0.25%), phenol red | Thermo Fisher | Catalog no. 25200072 |
| 6.5 mm Transwell with 3.0 µm Pore Polycarbonate Membrane Insert | Corning | Catalog no. 3415 |
| PrimeScript RT reagent kit with gDNA eraser kit | TaKaRa | Catalog no. RR047A |
| PowerUp SYBR Green Master Mix | Thermo Fisher | Catalog no. A25742 |
| ProLong Gold Antifade Mountant | Thermo Fisher | Catalog no. P36934 |
| 1x Dulbecco's Phosphate Buffered Saline (DPBS) | Thermo Fisher/Gibco | Catalog no. 21600010 |
| Carboxymethylcellulose (CMC) medium viscosity | Sigma | Catalog no. C4888 |
| Methanol | Merck | Catalog no. SB0SF70138 |
| Paraformaldehyde | Sigma-Aldrich | Catalog no. P6148 |
| HEPES | HiMedia | Catalog no. MB016 |
| 2019-nCoV CDC Probe and Primer kit for SARS-CoV-2 | Biosearch Technologies | Catalog no. KIT-nCoV-PP1-1000 |
| TaqMan RNase P Assay, VIC dye/QSY probe | Thermo Fisher | Catalog no: A30064 |
| Protease inhibitor cocktail (PIC) | Merck | Catalog no. 11836170001 |
| Phenylmethylsulfonyl fluoride  (PMSF) | Sigma-Aldrich | Catalog no. P7626 |
| PVDF membrane | GE Healthcare Life Sciences | Catalog no. 10600023 |
| RNA Isolation kit | Macherey-Nagel | Catalog no. 740955.250 |
| CellTiter-Glo assay kit | Promega | Catalog no. 7571 |
| Quant-iT PicoGreen dsDNA Assay Kit | Thermo-Fisher | Catalog no. 7589 |
|  |  |  |
| **Cell lines** |  |  |
| Vero E6 | ECACC | Catalog no. 85020206 |
| Calu-3 cells | ATCC | Catalog no. ATCC-HTB-55 |
|  |  |  |
| **Software and Algorithms** |  |  |
| Prism (9.1.0) | GraphPad |  |
| cellSens | Olympus |  |
| AID EliSpot 8.0 iSpot software | AID |  |
| Gen5 | BioTek |  |
| SoftMax pro GXP 7.1 | Molecular Devices |  |
|  |  |  |
| **Primers** |  |  |
| **Gene** | **Forward primer (5’-3’)** | **Reverse primer (5’-3’)** |
| *GAPDH* | CCACTCCTCCACCTTTGAC | ACCCTGTTGCTGTAGCCA |
| *IFN-β* | AAACTCATGAGCAGTCTGCA | AGGAGATCTTCAGTTTCGGAGG |
| *IFN-λ1* | GGTGACTTTGGTGCTAGGCT | TGAGTGACTCTTCCAAGGCG |
| *ISG15* | AGATCACCCAGAAGATCG | TGTTATTCCTCACCAGGATG |
| *OAS1* | TTCAGCGAGCTGCAGAGAAA | AAGAGCATAGAGAGGGGGCA |
