# Supplementary material for: BA.1, BA.2 and BA.2.75 variants show comparable replication kinetics, reduced impact on epithelial barrier and elicit cross-neutralizing antibodies
Source: PLoS Pathog. 2023 Feb 24;19(2):e1011196. doi: 10.1371/journal.ppat.1011196 (PMC9994724; doi:10.1371/journal.ppat.1011196)
Supplement: S1 Methods — (DOCX) [file ppat.1011196.s002.docx]

**SUPPORTING METHODS**

**Whole genome sequencing of SARS-CoV-2 using Nanopore platform**

Genome sequencing was performed according to Oxford Nanopore Technology (ONT) library preparation protocol- PCR tiling of SARS-CoV-2 virus with Rapid barcoding (Version: PCTR_9125_v110_revB_24Mar2021), commonly known as the midnight protocol. 50 ng of the extracted RNA from nasopharyngeal samples was reverse transcribed into cDNA using LunaScript RT SuperMix, comprising of random hexamer and oligo-dT primers, dNTPs, Murine RNase Inhibitor, and Reverse Transcriptase. The cDNA-RNA hybrid was used to amplify SARS-CoV-2 genome with rapid barcoding primers (IDT Product number: 10007184) and Q5 High-Fidelity 2X master mix (New England Biolabs, Cat. No. M0494S). The primer pools consisted of 30 and 28 primers each, that were designed specifically to produce 1200 bp amplicons with an approximate overlap of 20 bp. Targeted amplification, using multiplex PCR, was performed for each SARS-CoV-2 positive sample, separately for both the primer pools. The amplified products for both the primer pools were then combined and ligated to unique barcode sequences (SQK-RBK110.96). The barcode ligation step required a Transposase which simultaneously fragmented 1200 bp long amplicons and added barcode sequences to the cleaved ends. The barcoded library was pooled and then purified using SPRI beads. Tagging of unique barcode to each sample enabled pooling of multiple samples for sequencing together in the same library. The pooled barcoded library was quantified using Qubit dsDNA HS Assay kit. Finally, 800 ng of the library was then ligated with adapter protein and loaded on MinION Mk1B or MinION Mk1C platform.

**Nanopore Analysis Method**

The ARTIC end-to-end pipeline was used for the analysis of ONT MinION raw fast5 files up to variant calling. Raw fast5 files of samples was base called and demultiplexed using barcode kits information SQK-RBK110-96 with Guppy base caller that uses the base calling algorithms of Oxford Nanopore Technologies (Nanopore Community) with phred quality cut-off score >7 on GPU-linux accelerated computing machine. Reads having Phred quality score less than 7 were discarded to filter the low-quality reads. The resultant demultiplexed fastq were normalized by read length using the 1200 bp amplicon sets for further downstream analysis and aligned to the SARS-CoV-2 reference (MN908947.3) using the aligner Minimap2. Nanopolish were used to index raw fast5 files for variant calling from the minimap output files. To create consensus fasta, bcftools was used with normalized minimap2 output. Further, the assembled SARS-CoV-2 genomes were assigned lineages using the package Phylogenetic Assignment of Named Global Outbreak LINeages (PANGOLIN).

**Cell viability assay**

Cell viability assay was performed using CellTiter-Glo Assay kit as per the manufacturer’s instructions. This method determines the number of viable cells in culture based on quantification of the ATP present, in metabolically active cells. Briefly, Calu-3 cells were seeded at a density of 50,000 in 48-well plate and grown for 48 h. Cells were treated with indicated metal ions at a concentration of 50 µM in a total volume of 300 µl serum-free growth medium. After 24 h post-treatment, the assay was performed. 200 µl of medium was discarded from each well.100 µl of CellTiter-Glo reagent was added to the 100 µl of medium containing cells in each well. The contents were mixed for 2 min on rocker for cell lysis. The plate was incubated at room temperature for 10 min. The homogeneous CellTiter-Glo reagent results in cell lysis and generates a luminescent signal proportional to the amount of ATP present. 100 µl of content from each well was transferred to opaque-walled multiwell plates. The luminescence was measured by microplate reader (Synergy HT - BioTek).
