# Supplementary figures and images for: BA.1, BA.2 and BA.2.75 variants show comparable replication kinetics, reduced impact on epithelial barrier and elicit cross-neutralizing antibodies
Source: PLoS Pathog. 2023 Feb 24;19(2):e1011196. doi: 10.1371/journal.ppat.1011196 (PMC9994724; doi:10.1371/journal.ppat.1011196)

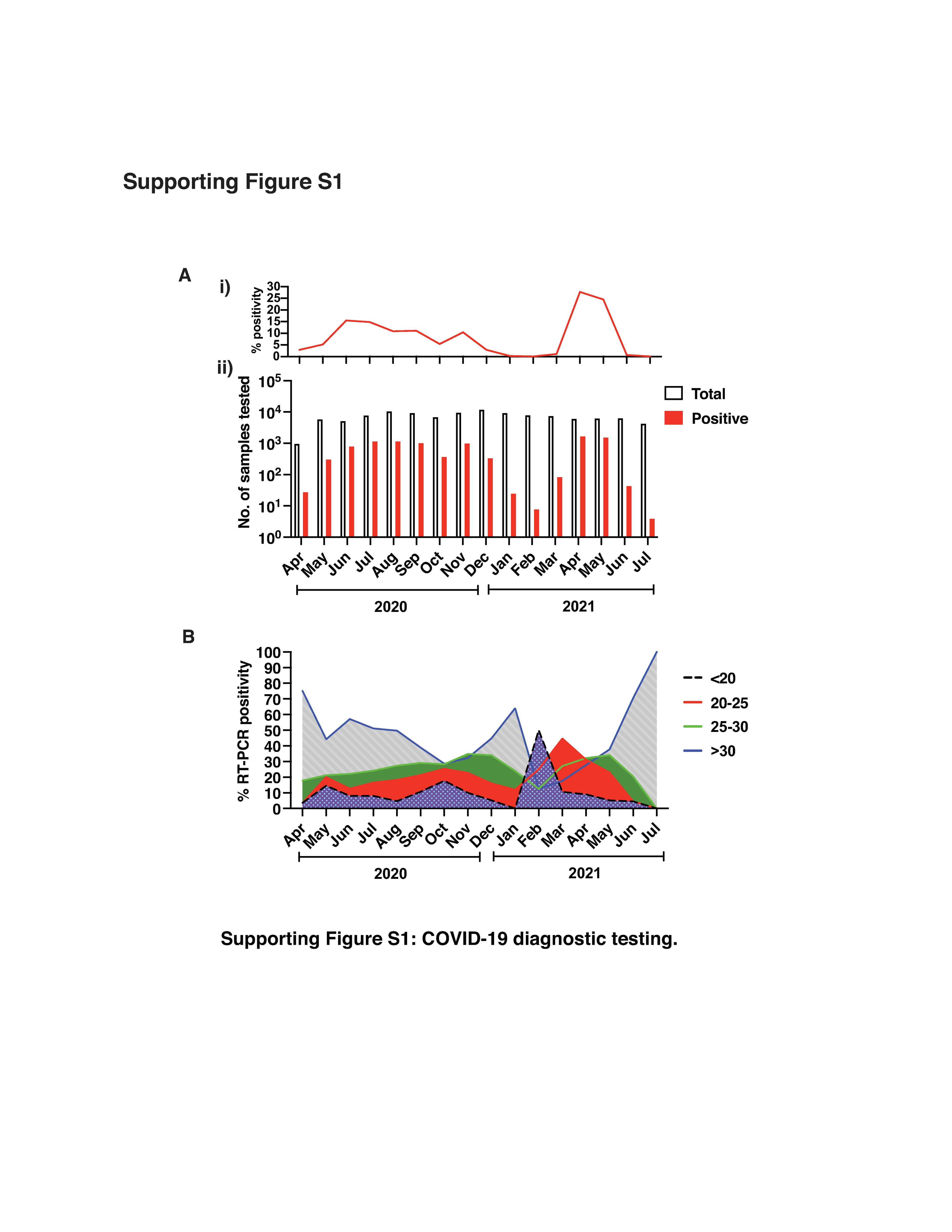

Supplement: S1 Fig — (A) NP/OP samples were tested by RT-PCR to diagnose COVID-19 infection and % positivity (i) and number of positive cases relative to the total number of samples tested (ii) from April 2020 to July 2021 is shown. (B) Threshold cycle (Ct) values of RNA samples tested for COVID-19 during the same period is shown and the values are segregated into four categories to indicate very high (< 20 Ct), high (20–25 Ct), moderate (25–30 Ct) and low (>30 Ct) viral burden in the first sample collected for diagnosis. (TIF) [file ppat.1011196.s005.tif]

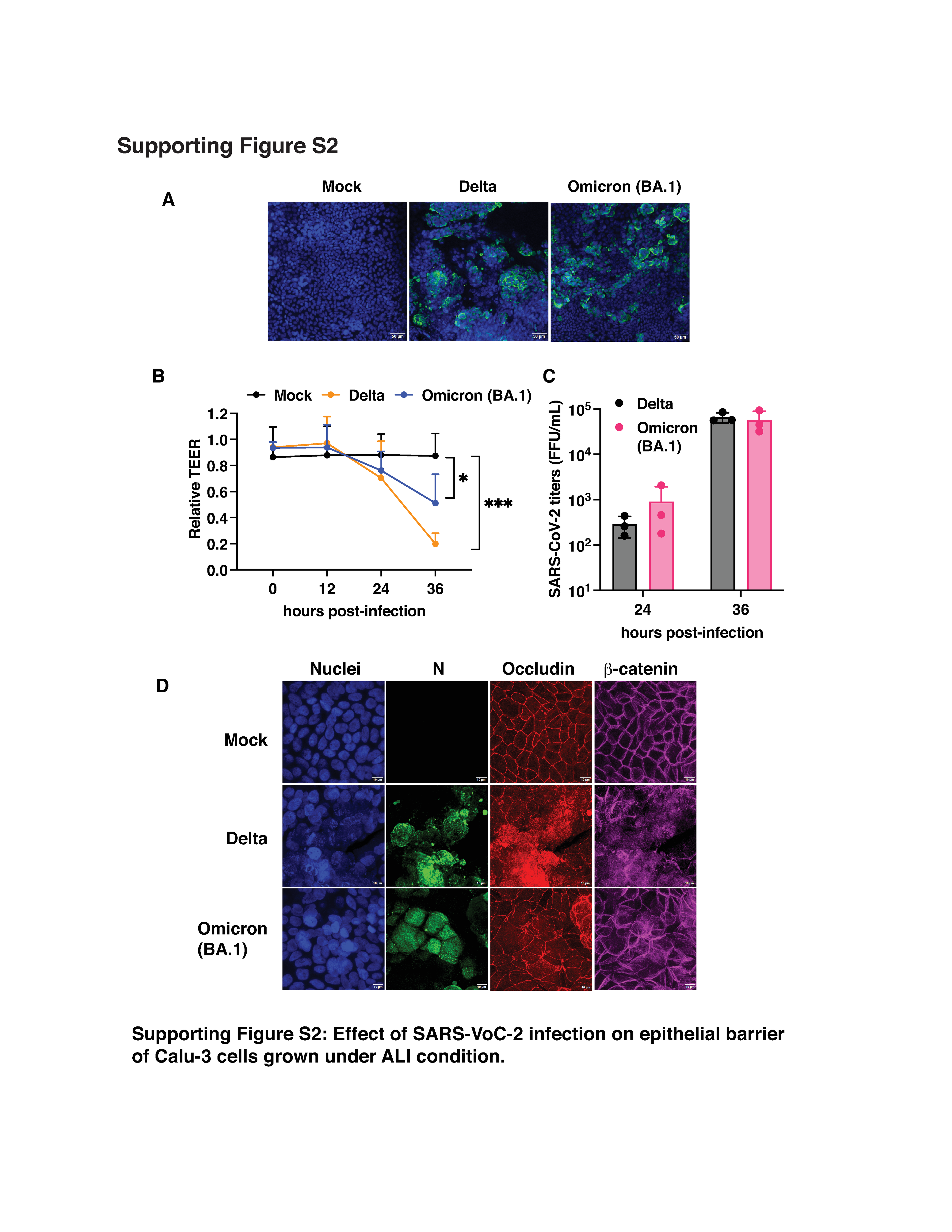

Supplement: S2 Fig — Calu-3 cells were grown on transwell inserts under air-liquid interface (ALI) conditions. Cells were infected with Delta and Omicron variants at 0.3 MOI. At 36 h pi, cells were fixed and stained with SARS-CoV-2 nucleocapsid antibody followed by Alexa Fluor 488-conjugated secondary antibodies for visualization. Nuclei were stained with DAPI. Images were captured at 20X magnification. Images were analyzed using cellSens software and Z-projection images with maximum intensity are shown in the figure. Scale bar is 50 μM. (B) Graph indicates TEER values relative to mock infection after infection at indicated time points from two independent experiments. (Mean and error with range). Statistical significance was estimated by two-way ANOVA with Tukey’s multiple comparisons test. (C) Viral titers were measured in supernatants by focus-forming units. Error bars represent (Mean ± SD) (D) At 36 h pi, cells were fixed and stained with occludin, β-catenin and SARS-CoV-2 nucleocapsid antibody followed by Alexa Fluor dye-conjugated secondary antibodies for visualization. Nuclei were stained with DAPI. Images were captured at 100X magnification. Images were analyzed using cellSens software and Z-projection images with maximum intensity are shown in the figure. Scale bar is 10 μM. ns: non-significant, **** P<0.0001. (TIF) [file ppat.1011196.s006.tif]

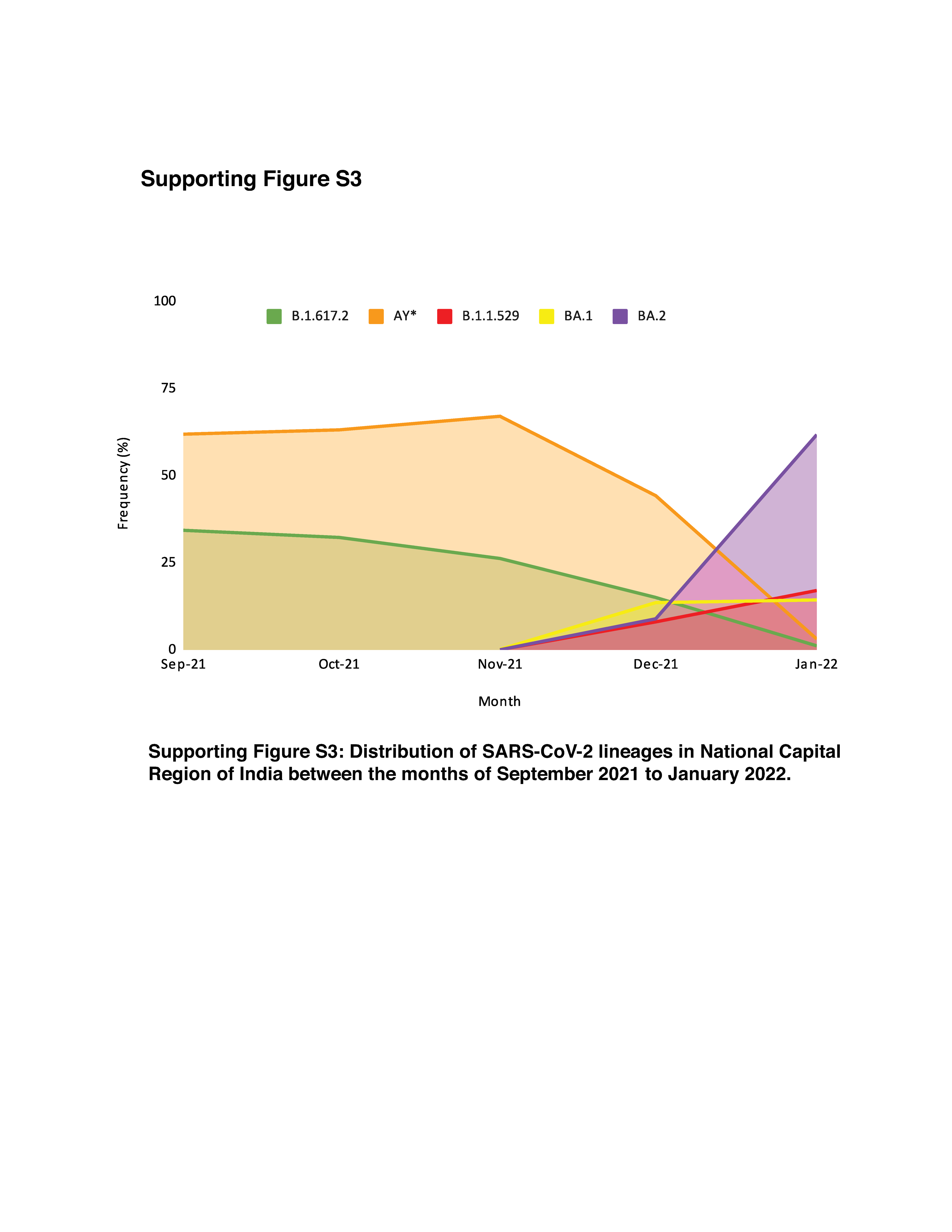

Supplement: S3 Fig — Whole genome sequencing of COVID-19 positive diagnostic samples for the indicated period. B.1.617.2 (Delta); AY.* (Delta plus); Omicron lineages (B.1.529, BA.1 and BA.2). (TIF) [file ppat.1011196.s007.tif]

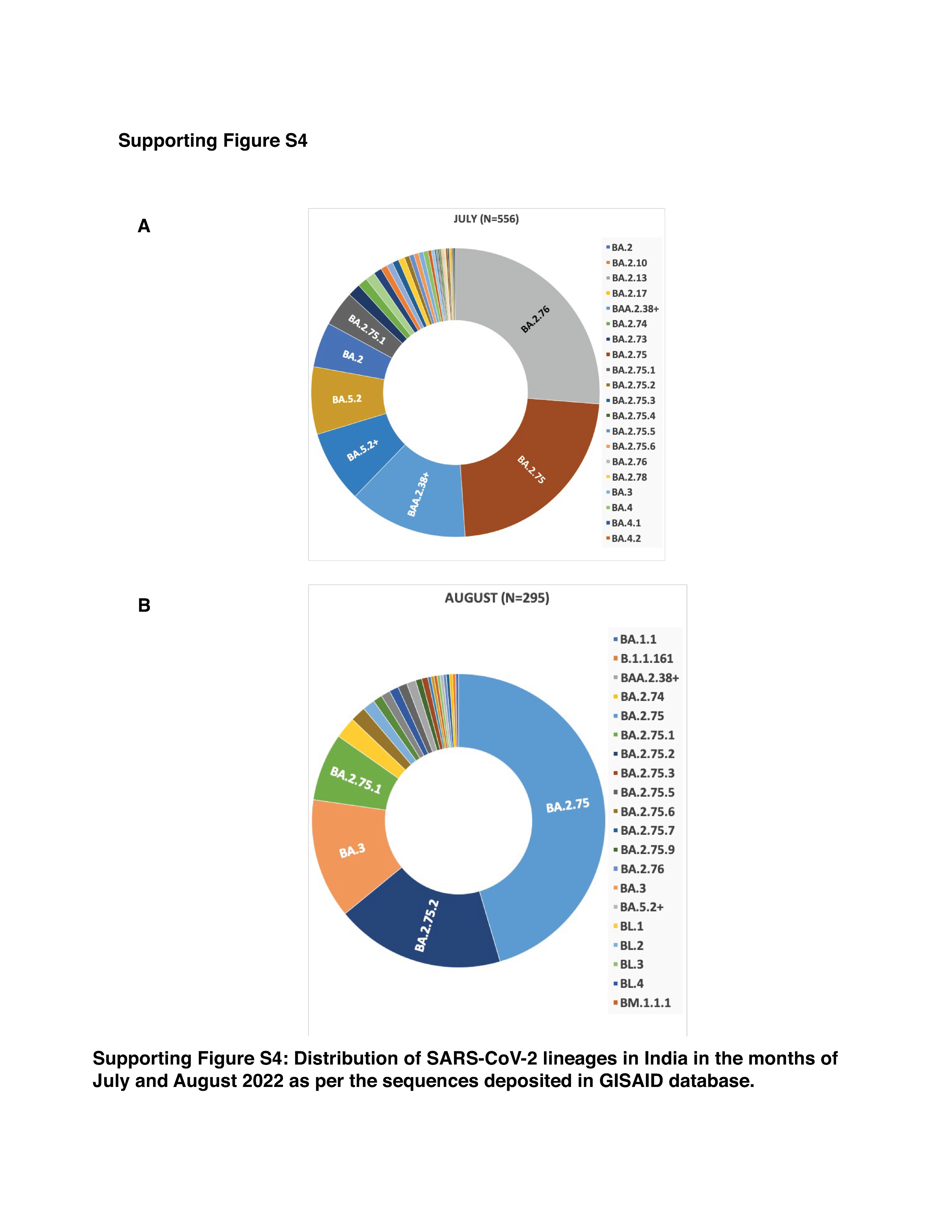

Supplement: S4 Fig — Virus circulation in the months of (A) July and (B) August 2022 as per the sequences deposited in GISAID database. (TIF) [file ppat.1011196.s008.tif]

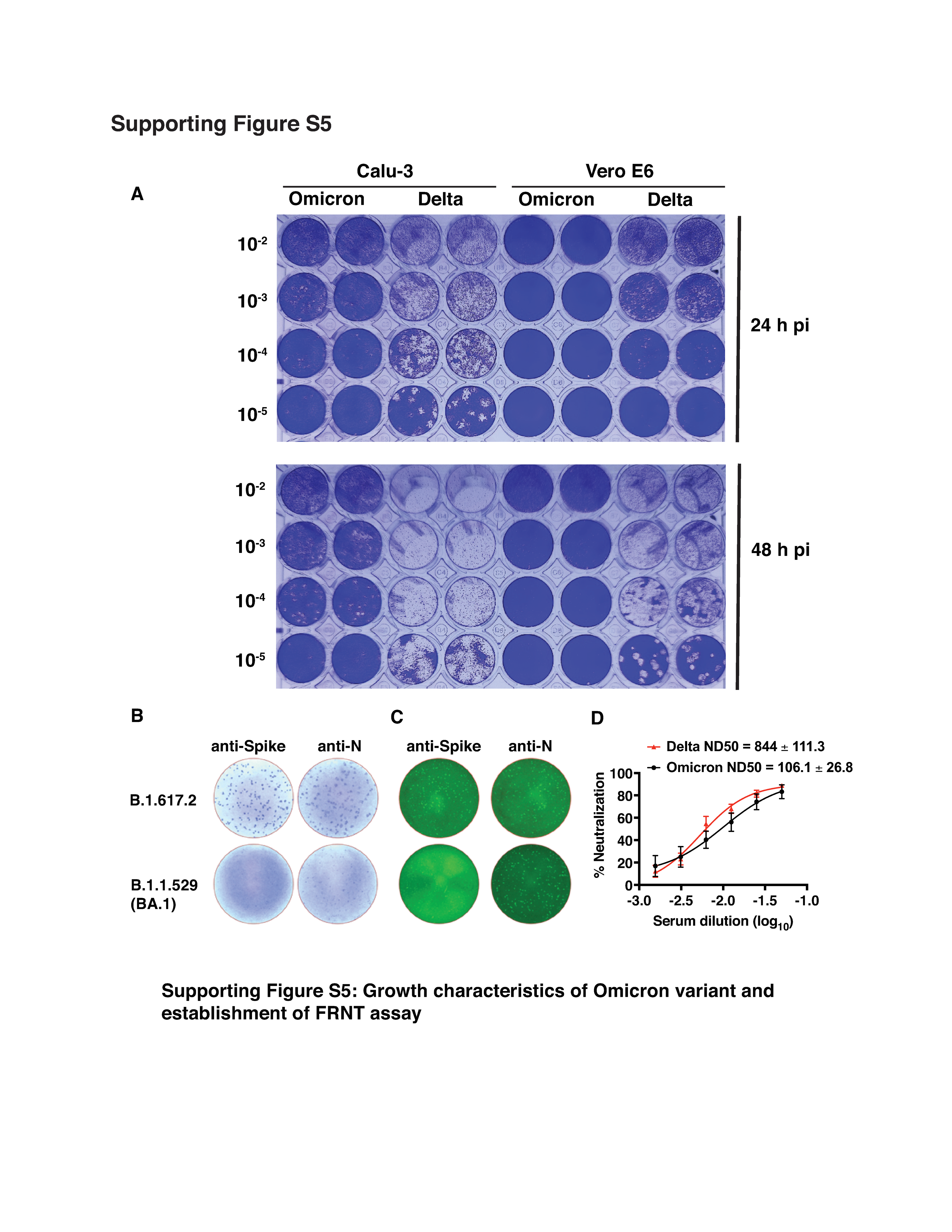

Supplement: S5 Fig — (A) Calu-3 or Vero E6 cells were incubated with 10-fold serial dilution of Omicron (BA.1) variant to determine virus titers by plaque assay. Plates were fixed at 24 and 48 h pi and stained with crystal violet. (B) Vero E6 cells were infected with a pre-determined dilution of Omicron variant for focus-forming unit assay using anti-spike and anti-nucleocapsid antibodies followed by HRP-conjugated secondary antibody. Foci were developed using TrueBlue substrate. (C) Vero E6 cells were infected with a pre-determined dilution of Omicron variant for focus-forming unit assay using anti-spike and anti-nucleocapsid antibodies followed by Alexa488-conjugated secondary antibody. Foci were visualized under fluorescence channel in the reader. (D) FRNT assay using fluorescence method to determine the neutralization titers of antibodies against the Delta and Omicron variants. 50% neutralization titer of antibodies (NT50) is given from at least five experimental replicates (Mean ± SD). (TIF) [file ppat.1011196.s009.tif]

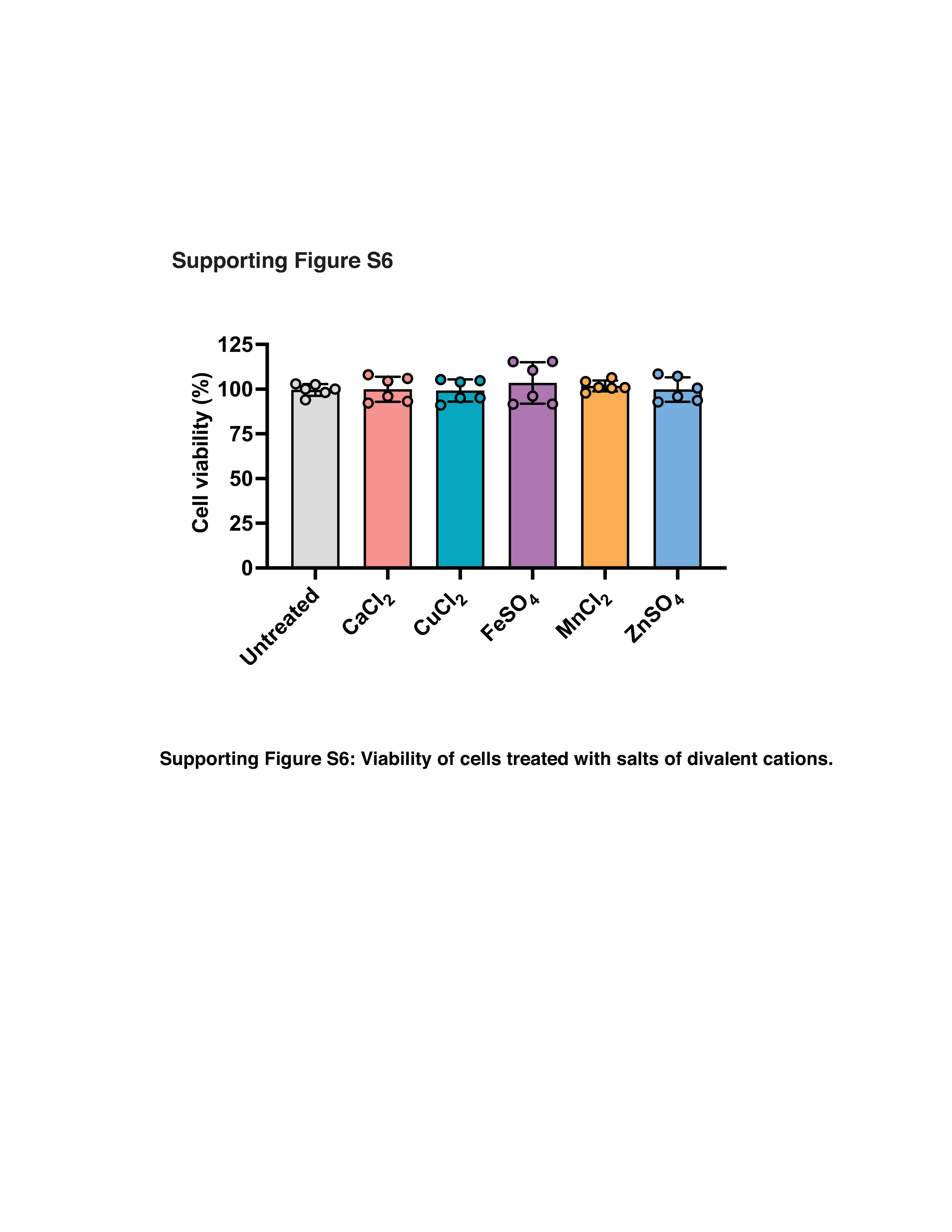

Supplement: S6 Fig — Calu-3 cells were treated with indicated salts at a concentration of 50 μM for 24 h. Cell viability assay was performed using CellTiter-Glo luminescent cell viability assay. Data from two experiments are presented as Mean + SD. (TIF) [file ppat.1011196.s010.tif]
